# How Adaptation of the Brain to Alcohol Leads to Dependence: A Pharmacological Perspective

**Published:** 2008

**Authors:** Peter Clapp, Sanjiv V. Bhave, Paula L. Hoffman

**Keywords:** Alcohol dependence, alcohol and other drug (AOD) dependence (AODD), addiction, neurobiology, neuroplasticity, neuroadaptation, brain, craving, withdrawal, relapse, neurotransmission, neurotransmitters, glutamate, glutamate receptors, glutamate systems, γ–aminobutyric acid (GABA), GABA systems, dopamine, serotonin, signaling molecules, endogenous opioids, opioid systems, corticotrophin-releasing factor (CRF), animal models, human studies

## Abstract

The development of alcohol dependence is posited to involve numerous changes in brain chemistry (i.e., neurotransmission) that lead to physiological signs of withdrawal upon abstinence from alcohol as well as promote vulnerability to relapse in dependent people. These neuroadaptive changes often occur in those brain neurotransmission systems that are most sensitive to the acute, initial effects of alcohol and/or contribute to a person’s initial alcohol consumption. Studies of these neuroadaptive changes have been aided by the development of animal models of alcohol dependence, withdrawal, and relapse behavior. These animal models, as well as findings obtained in humans, have shed light on the effects that acute and chronic alcohol exposure have on signaling systems involving the neurotransmitters glutamate, γ-aminobutyric acid (GABA), dopamine, and serotonin, as well as on other signaling molecules, including endogenous opioids and corticotrophin-releasing factor (CRF). Adaptation to chronic alcohol exposure by these systems has been associated with behavioral effects, such as changes in reinforcement, enhanced anxiety, and increased sensitivity to stress, all of which may contribute to relapse to drinking in abstinent alcoholics. Moreover, some of these systems are targets of currently available therapeutic agents for alcohol dependence.

The development of dependence on alcohol (as well as on other drugs of abuse) is posited to involve changes in brain chemistry that lead not only to signs of withdrawal upon abstention from alcohol (i.e., to physical or physiological dependence) (Ritzmann and Tabakoff 1976) but also, in humans, to the behaviors that define alcohol dependence, as described in the most recent edition of the *Diagnostic and Statistical Manual of Mental Disorders* (DSM–IV)^1^ (American Psychiatric Association 1994). It generally is thought that alcohol is consumed for its positive reinforcing effect—that is, to repeat the pleasurable experiences associated with initial alcohol consumption—and that chronic exposure to alcohol results in adaptations in brain function that eventually lead to dependence. This model leads to the question: What is the nature of the neurobiological and functional adaptations that result in the state of alcohol dependence?

In a recent review, Kalivas and O’Brien (2008) discussed the transition from “social” drug use to addiction, or dependence, in terms of transient and prolonged neuroplasticity. Neuroplasticity is defined as the brain’s ability to change and reorganize itself throughout life by forming new connections between nerve cells (i.e., neurons) and altering the activities of existing neurons. This ability allows the brain to compensate for injury or disease, to accommodate new experiences, and to adjust to new situations and changes in the environment (e.g., exposure to alcohol and other drugs [AODs]). With respect to AODs this means that even during the initial stages of AOD use, changes in brain chemistry occur that affect signaling molecules (i.e., neurotransmitters^2^), the proteins (i.e., receptors) that the neurotransmitters interact with, and various other molecules. These early changes, which are short lived and based on the initial effects of the particular drug in the brain, already may lead to signs of withdrawal when AOD use is stopped. Repeated exposure to the drug, however, induces longer-lasting changes in neuronal function that promote vulnerability to relapse behavior, which is related to habit formation. At this point, the drug-taking behavior is no longer under voluntary control.

When discussing the neurobiology that underlies the plastic changes associated with AOD use, Kalivas and O’Brien (2008) focused on the initial release of the neurotransmitter dopamine from cells in the brain region called the ventral tegmental area (VTA) that is induced by addictive drugs. The VTA is one of the components of a system of interconnected brain regions called the mesolimbic dopamine system. In this system, neurons whose cell bodies are located in the VTA, extend long “arms” (i.e., axons) to various other brain regions, most prominently the nucleus accumbens (NAc) and the prefrontal cortex (see figure 1). When activated, these neurons release dopamine that acts on other neurons in the NAc and prefrontal cortex. For many years, researchers thought that this dopamine release mediates positive reinforcing properties of AODs or other stimuli. More recently, it has been proposed that the dopamine release, particularly in the NAc, signals the importance (i.e., salience) (Iversen and Iversen 2007) of the stimulus to the individual. In either case, dopamine release in the mesolimbic system (e.g., NAc) likely is critical for the drive to ingest AODs. For example, Kalivas and O’Brien (2008) postulate that the released dopamine promotes neuroplasticity in the mesolimbic system through the activation of certain signaling pathways that ultimately alter gene expression. Such changes in gene expression may be associated with the transition from social drug use to relapsing drug use.

Signaling systems using the neurotransmitter glutamate also may undergo adaptive changes that contribute to AOD dependence. According to Kalivas and O’Brien (2008), adaptive changes in glutamate-using (i.e., glutamatergic) systems that transmit signals from various brain regions (e.g., the cortex, amygdala, and hippocampus) to the striatum are responsible for compulsive drug-seeking behavior in dependent people. The investigators cite evidence from human and animal studies suggesting that these neurochemical changes, as well as morphological changes, underlie a (mal)adaptive neuroplasticity that enhances the response to the addictive drug, or to cues associated with drug administration, while reducing the response to “normal” biologically rewarding stimuli. Together, these changes in the dopamine and glutamate systems may be the core changes that are the basis for the development of dependence on any drug.

In addition, researchers have investigated the long-lasting plasticity that specifically contributes to alcohol dependence. To this end, investigators have determined which neuronal systems initially are most sensitive to alcohol’s effects and/or play a role in voluntary alcohol consumption. Subsequently, they examined adaptations in these systems that can be observed after prolonged or chronic intermittent exposure to alcohol. Like other drugs of abuse, alcohol initially increases dopamine release in the mesolimbic system. Unlike most other addictive drugs, however, alcohol lacks a specific “receptor” in the brain.^3^ Instead, the effects of beverage alcohol (i.e., ethanol) on dopamine release may result from direct effects on the firing of dopamine neurons in the VTA and/or be mediated through interactions with other signaling systems, such as those using the neurotransmitters glutamate, γ-aminobutyric acid (GABA), and serotonin, as well as through interactions with the opioid and cannabinoid systems (see below).

Some of the adaptive changes caused by chronic alcohol exposure and acute withdrawal, such as decreased dopamine release in the mesolimbic system and striatum and increased glutamate transmission (e.g., Rossetti et al. 1999; Weiss et al. 1996), are similar to those leading to dependence on other drugs. Other changes, however, such as those involving the GABA system or a molecule called corticotrophin releasing factor (CRF) (which is involved in the brain’s stress response system), appear to be associated more specifically with acute alcohol withdrawal. These changes contribute to the anxiety-inducing (i.e., anxiogenic) and aversive effects of alcohol withdrawal and can persist over long periods of abstinence from alcohol. Eventually, these adaptations may result in increased anxiety and sensitivity to stress, so that the dependent person wants to drink alcohol in order to ameliorate these negative emotional states (Valdez and Koob 2004). At this stage, alcohol no longer is ingested for its positive reinforcing effects, but for negative reinforcement—that is, to eliminate unpleasant sensations, such as anxiety. These adaptive neurochemical changes, as well as morphological changes in some brain regions,^4^ can contribute to relapse to drinking. In summary, it appears that both the core changes associated with AOD dependence and other more specific alcohol-induced changes contribute to alcohol dependence, making it a very heterogeneous phenomenon.

This review focuses on neuroadaptation to acute and chronic alcohol exposure in several neurotransmitter systems—most prominently the glutamate, opiate, and GABA systems. The CRF system, which is sensitive to alcohol’s acute and chronic effects and is an important mediator of stress and anxiety, also is discussed. Although many more signaling systems are in some way or other affected by alcohol (for information on some of these, see the sidebar “Other Brain Signaling Systems Involved in Alcohol Dependence”), the discussion emphasizes those systems whose function is affected by currently available medications used to treat alcohol dependence. This discussion also takes into consideration the role of reduced reinforcement, enhanced anxiety, and increased sensitivity to stress as contributors to relapse drinking in the context of the neurobiological changes observed in alcohol-dependent people. Much of this research has been done in animal models that are designed to reflect various aspects of alcohol dependence in humans. For more information on these models, see the sidebar “Animal Models Used to Study Neuroadaptation.”

## Glutamate Systems and Alcohol Dependence

Glutamate is the primary excitatory neurotransmitter in the central nervous system. When an electrical nerve signal arrives at the axon terminal of a signal-emitting (i.e., presynaptic) neuron, glutamate stored in that neuron is released into the small gap (i.e., synaptic cleft) that separates that neuron from the signal-receiving (i.e., postsynaptic) neuron. The glutamate then interacts with receptors on the surface of the postsynaptic neuron, thereby initiating changes in that neuron that culminate in the generation of a new nerve signal in that cell. (For more information on the structure of a synapse and the process of neurotransmission, see the sidebar “Signal Transmission in the Nervous System.”)

### Glutamate Receptors

There are two main types of glutamate receptors:

- Ionotropic glutamate receptors (iGluRs), which are found on minute protrusions (i.e., spines) on the dendrites of the postsynaptic cells and produce relatively fast actions, thereby mediating rapid neuronal responses.
- Metabotropic glutamate receptors (mGluRs), which are located in the membrane around the synapse (i.e., perisynaptic membranes) and generally produce slower and longer-lasting reactions at the synapse that have modulatory effects rather than generate new nerve signals.

#### Ionotropic Glutamate Receptors

There are three classes of iGluRs that mediate the transmission of fast, excitatory signals:^5^

- *N*-methyl-d-aspartate receptors (NMDARs);
- α-Amino-3-hydroxy-5-methylisoxa-zole-4-proprionic acid receptors (AMPARs); and
- Kainic acid receptors.

Each NMDA receptor consists of several subunits that together form a channel through the membrane. Researchers have identified one type of NR1 subunit, four types of NR2 subunits, and two types of NR3 subunits.^6^ Each NMDAR complex comprises at least one NR1 subunit and a combination of NR2 and possibly NR3 subunits that together form a channel through which positively charged ions (i.e., cations, such as calcium ions [Ca^2+^]) can pass when the receptor is activated (Paoletti and Neyton 2007). Among these subunits, the NR2 subunits have a regulatory function by influencing agonist affinity^7^ as well as the rate at which the channel is activated and inactivated (Krupp et al. 1996; Laube et al. 2004).

When glutamate is released into the synapse, it can activate both AMPA and NMDA receptors (see figure 2A). AMPARs mediate the fast transmission of excitatory signals. Activation of AMPARs by glutamate allows for rapid cation (Na^+^) influx into the cell. This reduces the difference in electric charge between the cell’s inside and outside (i.e., the electric potential, measured in millivolts). A decrease in this electric potential is known as depolarization. When the cell is depolarized by activation of AMPARs, glutamate also can activate NMDARs. The activation of NMDARs by glutamate (and by the coagonist, glycine) allows additional Na^+^ and Ca^2+^ to enter the cell. These changes also open voltage-gated calcium channels in the postsynaptic membrane. As a result, an electrical signal (i.e., action potential) is generated that can be further transmitted throughout the cell to the axon. In addition, the increase in Ca^2+^ in the cell activates second messenger signaling pathways, including one involving a molecule called protein kinase A (PKA), and other kinases. These actions can have long-lasting effects, and NMDARs have been implicated in the generation of synapses in the developing brain (i.e., synaptic development), the ability to detect and integrate signals that occur simultaneously at the presynaptic terminal and postsynaptic membrane (i.e., coincidence detection), and long-lasting enhancement or reduction of neuronal activity (i.e., long-term potentiation and long-term depression) that are important for inducing neuroplasticity (Castellano et al. 2001). AMPARs also play an important role in neuroplasticity. Importantly, the location of AMPA receptors at the synapse is not fixed, and these receptors can be transported to and away from the postsynaptic membrane as needed. This trafficking of AMPARs plays an essential role in certain forms of experience-dependent plasticity and long-term changes in synaptic strength (Collingridge et al. 2004).

#### Metabotropic Glutamate Receptors

Similarly, there are several classes of mGluRs that mediate slow, modulatory transmission via activation of two classes of G-proteins:

- Group I receptors (i.e., mGluR1, mGluR5) activate a protein called Gαq.
- Group II receptors (i.e., mGluR2 and mGluR3) and Group III receptors (i.e., mGluR4, mGluR6, mGluR7, and mGluR8) activate a protein called Gαi (Conn and Pin 1997). Group II and Group III mGluRs also are present on the axon terminal of the presynaptic neuron. When they are activated by some of the glutamate released by the presynaptic neuron, they alter the presynaptic neuron’s activity so that further glutamate release is prevented (Schoepp 2001); this is called a negative feedback mechanism.

The mGluRs modulate glutamatergic neurotransmission by activating various signal transduction pathways. Although mGluRs do not cause membrane depolarization, they indirectly modulate excitatory transmission (Conn and Pin 1997). For example, Group I receptors (i.e., mGluR1 and mGluR5) can enhance NMDAR function by activating a signaling molecule called protein kinase C (PKC); moreover, these receptors are physically linked to the NMDA receptors (Fagni et al. 2000; Tu et al. 1999). Group II and Group III mGluRs can regulate glutamate release from the presynaptic axon by inhibiting certain enzymes essential for glutamate release (e.g., PKA). Moreover, Group II and III mGluRs can be located on adjacent neurons releasing the neurotransmitter GABA and help regulate the actions of those neurons (Schoepp 2001). Thus, mGluRs may serve to maintain the normal balance (i.e., homeostasis) of glutamatergic transmission and modulate aberrant changes in neuronal excitability.

### Effects of Acute Alcohol Exposure on the Glutamate System

Ethanol, at pharmacologically relevant concentrations, inhibits glutamatergic neurotransmission, primarily by acting on iGluRs, although some effects also have been noted on mGluRs (see figure 2B).^8^ Initial reports demonstrated that acute ethanol exposure inhibits NMDAR channel function in isolated neurons derived from the hippocampus and cerebellum (Hoffman et al. 1989; Lovinger et al. 1989). Subsequently, this observation has been repeated in many other systems, including the cerebral cortex, NAc, amygdala, and VTA (Hoffman 2003). These investigations further demonstrated that ethanol inhibition of NMDAR activation is non-competitive with glutamate—that is, the ethanol molecules do not compete with and displace glutamate molecules from the NMDAR; instead, receptor activation is reduced even though glutamate still binds to it. Ethanol also inhibits AMPAR channels by a non-competitive mechanism (Moykkynen et al. 2003). Because the influx of cations through iGluRs during excitatory neurotransmission is critical for inducing plasticity, it is not surprising that acute ethanol exposure negatively affects the induction of NMDA-dependent long-term potentiation as well as promotes long-term depression (Blitzer et al. 1990; Hendricson et al. 2002).

Not all iGluRs appear to be equally sensitive to acute ethanol exposure. Early work suggested that the specific NR2 subunits found in an NMDAR influence how sensitive the receptor is to acute inhibition by ethanol (Lovinger 1995). However, subsequent studies using laboratory-generated (i.e., recombinant) receptors of known subunit composition that were introduced into cells where they are not normally found (i.e., heterologous cells) demonstrated that differences in receptor sensitivity were small and inconsistent, depending on the cell type used (Blevins et al. 1997; Mirshahi et al. 1998). AMPARs, in contrast, do exhibit a significant difference in ethanol sensitivity that is subunit composition dependent. Thus, AMPARs comprising both GluR2 and GluR3 subunits, and receptors comprising only GluR3 subunits, were less sensitive to inhibition by ethanol than all other combinations tested (Akinshola et al. 2003).

**Other Brain-Signaling Systems Involved in Alcohol Dependence**

In addition to the neurotransmitter and signaling systems described in the main article that are affected by acute and chronic alcohol consumption and which exhibit neuroadaptation to prolonged presence of alcohol, several other brain-signaling systems also are affected by acute and chronic alcohol consumption. These include the serotonin and endogenous cannabinoid systems. Moreover, an intra-cellular signaling molecule called cyclic AMP response element-binding protein (CREB) helps mediate the production of many proteins and therefore plays a crucial role in the neuroadaptation in several signaling systems.

**Serotonin Systems**

In addition to the systems discussed above, other neurotransmitter and neuromodulator systems may have an important influence in alcohol dependence. For example, low activity of the neurotransmitter serotonin is associated with high alcohol intake (Petrakis 2006), and some selected lines of alcohol-preferring animals reportedly have lower brain levels of serotonin than their alcohol-nonpreferring counterparts (Casu et al. 2004; McBride et al. 1995; Murphy et al. 2002). Pharmacologic or genetic modulation of serotonin systems also has been found to alter ethanol consumption. Agents known as selective serotonin reuptake inhibitors (SSRIs), which increase extracellular serotonin levels in the brain by inhibiting molecules that transport serotonin back into the cells, reduce alcohol consumption in animals, with less consistent effects observed in humans (Maurel et al. 1999; Naranjo and Knoke 2001). Moreover, SSRIs had little effect on ethanol consumption in mice lacking the serotonin transporter (Boyce-Rustay et al. 2006).

Many studies have analyzed the effects of alcohol on serotonin-mediated neurotransmission in the brain. These studies found that serotonin transmission is increased after acute alcohol administration and reduced during alcohol withdrawal (Tabakoff and Hoffman 1977). Decreased serotonin neurotransmission in dependent animals may be associated with relapse drinking. For example, when serotonin neurotransmission was inhibited by injecting a γ-aminobutyric acid (GABA_A_) receptor agonist into the brainstem (which reduces the activity of serotonin-releasing neurons), alcohol drinking in alcohol-withdrawn rats was reinstated (Lê et al. 2008).

There are numerous subtypes of serotonin receptors (Hoyer et al. 2002), and it is possible that serotonin can affect alcohol drinking by activating specific receptors. For example, activation of 5-HT2C or 5-HT1A serotonin receptors reduces alcohol consumption (Long et al. 1996; Tomkins et al. 2002). However, both increases and decreases in 5-HT1B receptor production can increase ethanol consumption (Hoplight et al. 2006; Risinger et al. 1999), with overproduction of the 5-HT1B receptor reportedly producing the most significant changes. Conversely, inhibition of the 5-HT3 receptor substantially reduced alcohol consumption (Hodge et al. 2004). In fact, the 5-HT3 receptor antagonist ondansetron has had some success in reducing alcohol consumption and increasing abstinence in alcohol-dependent people (Ait-Daoud et al. 2001; Johnson et al. 2000; Kranzler et al. 2003), as has the 5-HT1A partial agonist buspirone (Kranzler et al. 1994). These studies demonstrate that the function and localization of the various types of serotonin receptors determine their role in modulating alcohol consumption.

**Endogenous Cannabinoids**

Researchers also are exploring the interaction of ethanol with endogenous cannabinoids—substances naturally produced in the body that have similar effects to cannabis and related drugs—and the cannabinoid CB1 receptor. Endogenous cannabinoids appear to be involved in alcohol-induced activation of ventral tegmental area (VTA) neurons, possibly through interactions with opioid systems (Manzanares et al. 2005; Perra et al. 2005). Chronic alcohol exposure alters both the synthesis of endogenous cannabinoids and the characteristics of CB1 receptors (Vinod and Hungund 2005). In addition, alcohol consumption and alcohol-induced mesolimbic dopamine release were reduced in mice lacking the CB1 receptor (Hungund et al. 2003). Finally, a CB1 receptor antagonist reduced cue-induced alcohol reinstatement and the alcohol deprivation effect in rats (Colombo et al. 2007). However, clinical studies testing a CB1 receptor antagonist, rimonabant, for weight loss have noted side effects of severe depression, anxiety, and increased risk of suicide, which could limit the use of such antagonists.

**CREB Protein**

Researchers also are investigating the role of a molecule called CREB, which is not a neurotransmitter but is found inside of cells. It is involved in the cell’s response to the second-messenger molecule cyclic AMP, which, as described in the main article, helps mediate the activity of many metabotropic neurotransmitter receptors. Low activity of CREB in certain regions of the brain (i.e., the amygdala) is associated with anxiety, including alcohol withdrawal-induced anxiety, and increased alcohol consumption (Pandey 2004; Pandey et al. 2005). CREB is a protein that can bind to DNA and affect the production of other proteins. For proteins like CREB to bind to DNA, however, the structure of the DNA, which is called chromatin, must be “opened up.” Very recently, changes in brain (amygdala) chromatin remodeling, which is important for binding of proteins like CREB and subsequent transcriptional processes, were found to be associated with alcohol withdrawal-induced anxiety-like behaviors in rats (Pandey et al. 2008). These findings suggest a cell, signaling mechanism by which changes in various neurotransmitters that influence cAMP levels could result in the same effects (i.e., withdrawal-induced anxiety and relapse drinking).

ReferencesAit-DaoudNJohnsonBAJavorsMCombining ondansetron and naltrexone treats biological alcoholics: Corroboration of self-reported drinking by serum carbohydrate deficient transferrin, a biomarkerAlcoholism: Clinical and Experimental Research25847849200111410720Boyce-RustayJMWiedholzLMMillsteinRAEthanol-related behaviors in serotonin transporter knockout miceAlcoholism: Clinical and Experimental Research301957196520061711795910.1111/j.1530-0277.2006.00241.xCasuMAPisuCLobinaCPaniLImmunocytochemical study of the forebrain serotonergic innervation in Sardinian alcohol-preferring ratsPsychopharmacology (Berlin)17234135120041463471710.1007/s00213-003-1663-zColomboGOrruALaiPThe cannabinoid CB1 receptor antagonist, rimonabant, as a promising pharmacotherapy for alcohol dependence: Preclinical evidenceMolecular Neurobiology3610211220071795265510.1007/s12035-007-0017-yHodgeCWKelleySPBrattAM5-HT(3A) receptor subunit is required for 5-HT3 antagonist-induced reductions in alcohol drinkingNeuropsychopharmacology291807181320041516215810.1038/sj.npp.1300498HoplightBJSandygrenNANeumaierJFIncreased expression of 5-HT1B receptors in rat nucleus accumbens via virally mediated gene transfer increases voluntary alcohol consumptionAlcohol38737920061683985310.1016/j.alcohol.2006.04.003HoyerDHannonJPMartinGRMolecular, pharmacological and functional diversity of 5-HT receptorsPharmacology, Biochemistry and Behavior7153355420021188854610.1016/s0091-3057(01)00746-8HungundBLSzakallIAdamACannabinoid CB1 receptor knockout mice exhibit markedly reduced voluntary alcohol consumption and lack alcohol-induced dopamine release in the nucleus accumbensJournal of Neurochemistry8469870420031256251410.1046/j.1471-4159.2003.01576.xJohnsonBARoacheJDJavorsMAOndansetron for reduction of drinking among biologically predisposed alcoholic patients: A randomized controlled trialJAMA: Journal of the American Medical Association28496397120001094464110.1001/jama.284.8.963KranzlerHRBurlesonJADel BocaFKBuspirone treatment of anxious alcoholics: A placebo-controlled trialArchives of General Psychiatry517207311994808034910.1001/archpsyc.1994.03950090052008KranzlerHRPierucci-LaghaAFeinnRHernandez-AvilaCEffects of ondansetron in early- versus late-onset alcoholics: A prospective, open-label studyAlcoholism: Clinical and Experimental Research271150115520031287892110.1097/01.ALC.0000075547.77464.76LêADFunkDHardingSIntra-median raphe nucleus (MRN) infusions of muscimol, a GABA-A receptor agonist, reinstate alcohol seeking in rats: Role of impulsivity and rewardPsychopharmacology (Berlin)19560561520081789138110.1007/s00213-007-0943-4LongTAKalmusGWBjorkAMyersRDAlcohol intake in high alcohol drinking (HAD) rats is suppressed by FG5865, a novel 5-HT1A agonist/5-HT2 antagonistPharmacology, Biochemistry and Behavior5333401996884845710.1016/0091-3057(95)00195-6ManzanaresJOrtizSOlivaJMInteractions between cannabinoid and opioid receptor systems in the mediation of ethanol effectsAlcohol and Alcoholism40253420051555045110.1093/alcalc/agh112MaurelSDe VryJSchreiberRComparison of the effects of the selective serotonin-reuptake inhibitors fluoxetine, paroxetine, citalopram and fluvoxamine in alcohol-preferring cAA ratsAlcohol1719520119991023116710.1016/s0741-8329(98)00046-9McBrideWJBodartBLumengLLiTKAssociation between low contents of dopamine and serotonin in the nucleus accumbens and high alcohol preferenceAlcoholism: Clinical and Experimental Research19142014221995874980410.1111/j.1530-0277.1995.tb01001.xMurphyJMStewartRBBellRLPhenotypic and genotypic characterization of the Indiana University rat lines selectively bred for high and low alcohol preferenceBehavior Genetics3236338820021240551710.1023/a:1020266306135NaranjoCAKnokeDMThe role of selective serotonin reuptake inhibitors in reducing alcohol consumptionJournal of Clinical Psychiatry62Suppl. 201825200111584871PandeySCThe gene transcription factor cyclic AMP-responsive element binding protein: Role in positive and negative affective states of alcohol addictionPharmacology & Therapeutics104475820041550090810.1016/j.pharmthera.2004.08.002PandeySCZhangHRoyAXuTDeficits in amygdaloid cAMP-responsive element-binding protein signaling play a role in genetic predisposition to anxiety and alcoholismJournal of Clinical Investigation1152762277320051620021010.1172/JCI24381PMC1236671PandeySCUgaleRZhangHBrain chromatin remodeling: A novel mechanism of alcoholismJournal of Neuroscience283729373720081838533110.1523/JNEUROSCI.5731-07.2008PMC6671100PerraSPillollaGMelisMInvolvement of the endogenous cannabinoid system in the effects of alcohol in the mesolimbic reward circuit: Electrophysiological evidence in vivoPsychopharmacology (Berlin)18336837720051622819410.1007/s00213-005-0195-0PetrakisILA rational approach to the pharmacotherapy of alcohol dependenceJournal of Clinical Psychopharmacology26Suppl. 1S31220061711495210.1097/01.jcp.0000248602.68607.81RisingerFODoanAMVickreyACOral operant ethanol self-administration in 5-HT1b knockout miceBehavioral Brain Research10221121519991040302810.1016/s0166-4328(99)00012-1TabakoffBHoffmanPLTolerance and physical pependence: Noradrenergic and serotonergic correlatesSeixasFACurrents in AlcoholismGrune & Stratton, Inc1977123137TomkinsDMJoharchiNTampakerasMAn investigation of the role of 5-HT(2C) receptors in modifying ethanol self-administration behaviourPharmacology, Biochemistry and Behavior7173574420021188856510.1016/s0091-3057(01)00710-9VinodKYHungundBLEndocannabinoid lipids and mediated system: Implications for alcoholism and neuropsychiatric disordersLife Sciences771569158320051600547110.1016/j.lfs.2005.05.041

Many of the behavioral effects of acute ethanol exposure can be linked to effects on glutamatergic neuro-transmission. Pharmacological agents that, like ethanol, inhibit iGluR activity have ethanol-like discriminative stimulus properties^9^ in rats and, in some cases, make the animals even more sensitive to the locomotor stimulant effects of low doses of ethanol (Backstrom and Hyytia 2004; Butelman et al. 1993; Grant et al. 1991 Meyer and Phillips 2003). Similarly, in detoxified alcohol-dependent humans, NMDAR antagonists^10^ such as ketamine produce subjective intoxicating effects that resemble those of alcohol (Krystal et al. 1998). mGluRs also have been implicated in alcohol-related behaviors. In animal models, treatment with mGluR5 inhibitors reduced the rewarding effects of alcohol under certain experimental conditions, decreased alcohol consumption, and prevented alcohol-dependent changes in glutamate and dopamine release from NAc neurons (Hodge et al. 2006; Lominac et al. 2006). Moreover, mice that lack the gene for a protein which normally links Group I mGluRs and NMDARs in synaptic spines show reduced preference for alcohol (Szumlinski et al. 2005).

**Animal Models Used to Study Neuroadaptation**

Much of the work investigating the neurobiological changes produced by chronic alcohol exposure depends on the use of animal models. However, most of the human behaviors that define the DSM–IV diagnosis of alcohol dependence and which reflect essential characteristics of alcohol addiction (e.g., compulsive drug seeking and drug use, even in the face of negative health and social consequences) cannot be directly modeled in animals (Leshner 1997).

Another fundamental aspect of dependence in humans is the occurrence of relapse to alcohol and other drug (AOD) use during periods of protracted abstinence (Spanagel and Kiefer 2008). One key element of relapse is craving—that is, the desire to repeat the effect(s) of a previously experienced psychoactive substance (Spanagel and Holter 1999; Spanagel and Kiefer 2008). In a three-stage model of dependence, craving also has been conceptualized as the preoccupation/anticipation stage (Koob 2008).

Craving in humans is a somewhat controversial topic because it may define a physiological or subjective state that may or may not be a requisite for alcohol use or relapse (Spanagel and Holter 1999). In animals, researchers have developed an operational definition of craving that allows them to investigate the neurobiology of craving for AODs. According to this definition, craving is the “incentive motivation to self-administer a [psychoactive] drug which was previously consumed” (Markou et al. 1993, p.164). A key animal model that aims at measuring craving for alcohol (and other drugs) is the reinstatement model (de Wit and Stewart 1981; Shaham et al. 2003), which reflects alcohol-seeking behavior. In this model, an animal is trained to self-administer (i.e., work for) alcohol. In other words, alcohol serves as a reinforcer that motivates the animal to perform an operant response (e.g., pressing a lever to obtain alcohol). The animal then is tested under conditions where the alcohol is not available. The lack of the alcohol reinforcer causes the animal to stop its operant responding behavior, a process known as extinction. The extinguished behavior, however, can be reinstated by a cue that has previously been associated with alcohol (i.e., a conditioned stimulus), by stress, or by alcohol administration (Spanagel and Kiefer 2008). Under those conditions, the animal will work for alcohol even if no alcohol is provided. This model takes into account the findings that cues in the environment that previously have been associated with alcohol drinking as well as environmental factors such as stress, can trigger craving and relapse drinking in alcohol-dependent people (Walter et al. 2006). Different neurobiological pathways may underlie the various stimuli for reinstatement (e.g., Koob 2008; Vengeliene et al. 2008).

Another animal model of relapse behavior is the alcohol deprivation effect (Le Magnen 1960; Sinclair and Senter 1967; Sanchis-Segura and Spanagel 2006a), which may be related to the dysphoric effect associated with acute withdrawal. In the three-stage model of dependence, this is conceptualized as the withdrawal/negative-affect stage (Koob 2008). With this approach, mice and rats are chronically exposed to alcohol, followed by periods of abstinence. When alcohol is reintroduced under these conditions, the animals will drink substantially more than before the abstinence period. In a similar model, called withdrawal-induced drinking, mice are trained to self-administer alcohol, then exposed chronically and repeatedly to alcohol vapor, followed by periods of abstinence. After this treatment, the mice self-administer greater amounts of alcohol than before the chronic exposure and abstinence (Finn et al. 2007). These models may be similar to the alcohol-induced reinstatement model described above in that alcohol intake is stimulated by cues (e.g., odor) related to alcohol; however, they require a shorter abstinence period. As noted by Koob (2008), at least some neurobiological systems may be involved both in relapse associated with this acute withdrawal/negative affect stage of alcohol dependence and in craving and relapse during protracted abstinence.

Both the alcohol deprivation effect and the reinstatement of alcohol responding in animals can be reduced with pharmacological agents that have relatively modest effects in reducing relapse in alcohol-dependent people. Accordingly, both of these models can be used not only to test such therapeutic agents but also to understand the adaptive neurobiological changes that contribute to alcohol dependence. The two therapeutic agents currently used to reduce alcohol drinking in alcohol-dependent people are acamprosate (Campral^®^), which is thought to modulate the activity of the glutamate systems in brain, and naltrexone (Revia^®^), which acts on the brain’s opiate system (Spanagel and Kiefer 2008). The role of these systems in alcohol dependence is discussed in the main article.

Referencesde WitHStewartJReinstatement of cocaine-reinforced responding in the ratPsychopharmacology (Berlin)751341431981679860310.1007/BF00432175FinnDASnellingCFretwellAMIncreased drinking during withdrawal from intermittent ethanol exposure is blocked by the CRF receptor antagonist D-Phe-CRF(12–41)Alcoholism: Clinical and Experimental Research3193994920071740306810.1111/j.1530-0277.2007.00379.xKoobGFA role for brain stress systems in addictionNeuron59113420081861402610.1016/j.neuron.2008.06.012PMC2748830Le MagnenJ[Study of some factors associated with modifications of spontaneous ingestion of ethyl alcohol by the rat.]Journal de Physiologie (Paris)52873884196013759859LeshnerAIDrug abuse and addiction treatment research: The next generationArchives of General Psychiatry546916941997928350210.1001/archpsyc.1997.01830200015002MarkouAWeissFGoldLHAnimal models of drug cravingPsychopharmacology (Berlin)1121631821993787101610.1007/BF02244907Sanchis-SeguraCSpanagelRBehavioural assessment of drug reinforcement and addictive features in rodents: an overviewAddiction Biology1123820061675933310.1111/j.1369-1600.2006.00012.xShahamYShalevULuLThe reinstatement model of drug relapse: History, methodology and major findingsPsychopharmacology (Berlin)16832020031240210210.1007/s00213-002-1224-xSpanagelRHolterSMLong-term alcohol self-administration with repeated alcohol deprivation phases: An animal model of alcoholism?Alcohol and Alcoholism3423124319991034478310.1093/alcalc/34.2.231SpanagelRKieferFDrugs for relapse prevention of alcoholism: Ten years of progressTrends in Pharmacological Sciences2910911520081826266310.1016/j.tips.2007.12.005VengelieneVBachtelerDDanyszWSpanagelRThe role of the NMDA receptor in alcohol relapse: A pharmacological mapping study using the alcohol deprivation effectNeuropharmacology4882282920051582925410.1016/j.neuropharm.2005.01.002WalterMGerhardUDuersteler-MacFarlandKMSocial factors but not stress-coping styles predict relapse in detoxified alcoholicsNeuropsychobiology5410010620061710871010.1159/000096991

Acute ethanol exposure also exhibits presynaptic effects on glutamatergic signal transmission. In spinal moto-neurons of newborn rats, ethanol decreased the frequency of NMDAR-and AMPAR-dependent postsynaptic electrical signals (so-called excitatory postsynaptic currents [mEPSCs]), suggesting that ethanol inhibited glutamate release into the synapse (Ziskind-Conhaim et al. 2003). Similarly, acute ethanol exposure reduced the frequency and amplitude of NMDA-mediated mEPSCs in neurons in the NAc (Zhang et al. 2005). Such effects may be mediated by ethanol-sensitive mGluRs on presynaptic axon terminals. Other studies found that when presynaptic mGluR2/3 were inhibited, the acute sedative and hypnotic effects of ethanol in mice were reduced (Sharko and Hodge 2008). This finding suggests that ethanol promotes activation of these mGluRs.

### Effects of Chronic Alcohol Exposure on the Glutamate System

When glutamate receptors are inhibited for extended periods of time because of sustained ethanol exposure, the body tries to adapt to the chronic presence of ethanol and employs several mechanisms to maintain “normal” receptor activity even in the presence of ethanol (see figure 2C). For example, after long-term ethanol exposure, when ethanol has been eliminated from the cells, the function of NMDARs in cells of the cerebellum and cortex is found to be increased (i.e., there is a greater response to glutamate) (Ahern et al. 1994; Iorio et al. 1992). Moreover, after chronic ethanol exposure, the production of NMDAR subunits was increased in various brain regions of rodents (e.g., hippocampus, amygdala, and cerebral cortex), resulting in a greater number of receptor complexes (Floyd et al. 2003; Kalluri et al. 1998; Snell et al. 1996). In cortical tissue obtained from ethanol-dependent patients after death, binding of ligands^11^ to the NMDARs was increased (Freund and Anderson 1996). Finally, studies using neurons isolated from the hippocampus and grown in culture found that after chronic ethanol exposure more ions pass through the channel once it is opened (i.e., channel conductance is enhanced) and more NMDARs tend to cluster at the synapse. At the same time, the size of synaptic spines in these neurons is increased, further supporting the presence of additional NMDAR complexes (Carpenter-Hyland et al. 2004; Clapp et al. 2007).

The synaptic population of AMPARs also changes in response to prolonged ethanol exposure. For example, chronic ethanol treatment increased AMPAR-mediated Ca^2+^ flow into the neurons as well as production of GluR1 and GluR2/3 subunits in neuronal cultures and in some brain regions (Chandler et al. 1999; Dettmer et al. 2003). However, in contrast to the NMDARs, no increased synaptic clustering of AMPARs occurred in cultured hip-pocampal neurons chronically exposed to ethanol (Carpenter-Hyland et al. 2004). Finally, in rats subjected to chronic intermittent ethanol exposure (i.e., periods of alcohol exposure followed by periods of abstinence), AMPAR-mediated spontaneous EPSCs in tissue slices obtained from a part of the amygdala exhibited a higher frequency (suggesting increased glutamate release) and amplitude (Lack et al. 2007).

**Signal Transmission in the Nervous System**

The nerve cell (i.e., neuron) is the central component of the nervous system. It has three main structural features (see figure):

- The dendrites—thin branched fibers that extend from the cell body and receive signals from other cells. The dendrites often have minute protrusions (i.e., dendritic spines) that serve as the contact point (i.e., synapse) to other nerve cells.
- The cell body, which carries out the cell’s main cellular functions.
- The axon—a long, thin fiber that carries nerve impulses to other neurons.

**Figure:**
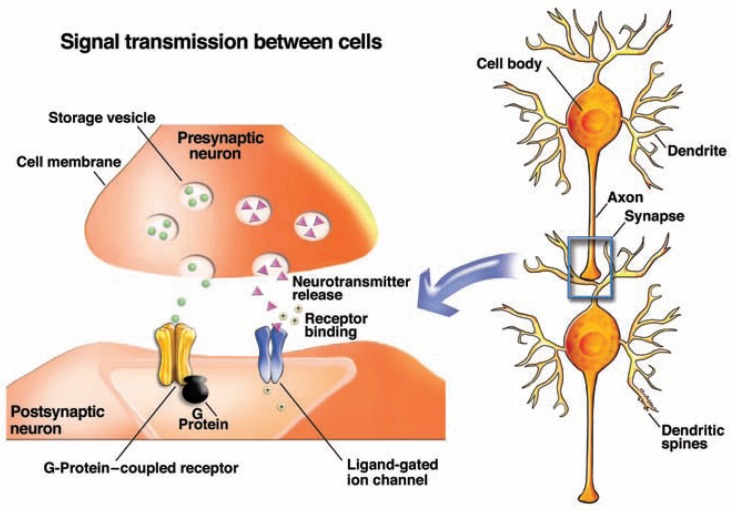

Information among neurons or between neurons and other types of cells is conveyed both electrically and chemically. Within a neuron, signals are passed on electrically, through the movement of an electrical impulse along the cell membrane. To transmit the information to other cells, the electrical signal is converted into a chemical signal conveyed by small molecules called neurotransmitters.

**Signal Transmission Within Neurons**

Electrical signal transmission within neurons is based on voltage differences (i.e., an electrical potential) between the inside and outside of the cell, which is created by the uneven distribution of positively and negatively charged ions. The most important of these ions are sodium (Na^+^), potassium (K^+^), calcium (Ca^2+^), and chloride (Cl^−^). To enter and exit the cell, the ions have to pass through specific protein channels in the cell’s membrane. These channels “open” or “close” in response to the binding of neurotransmitters (i.e., lig-and-gated channels) or to changes in the membrane’s potential (i.e., voltage-gated channels). When the channels open, the corresponding ions can enter or exit the cell, resulting in redistribution of the electrical charges that may decrease the membrane potential. This is known as depolarization. If depolarization exceeds a certain threshold, an electrical impulse (i.e., action potential) is generated that can travel along the neuron, toward the tip of the axon, where it is converted into a chemical signal.

**Signal Transmission Between Cells**

The axon tip of a signal-emitting, or presynaptic, neuron and the synaptic region of the signal-receiving, or postsynaptic, neuron are separated by a small gap (i.e., synaptic cleft). To allow the signal to cross this gap, the presynaptic neuron releases a neurotransmitter that can migrate across the synaptic cleft and interact with docking molecules (i.e., receptors) on the postsynaptic neuron. The neurotransmitter release is initiated by the arrival of an action potential at the axon tip. The resulting depolarization causes vesicles containing stored neurotransmitter molecules to fuse with the cell membrane and release their contents into the synaptic cleft. Each neuron produces and releases only one or a few types of neurotransmitters but carries receptors for several different types of neurotransmitters on its surface.

On the postsynaptic cell, the released neurotransmitter binds to its receptors, thereby triggering changes in the postsynaptic cell that either promote or inhibit the formation of new action potentials. Neurotransmitters whose binding to their receptors promotes the formation of a new action potential are called excitatory neurotransmitters; conversely, neurotransmitters whose binding to their receptors makes generation of a new action potential more difficult are called inhibitory neurotransmitters.

Neurotransmitter receptors also fall into two classes:

- Ionotropic receptors are ligand-gated channel receptors located directly at the synapse on the dendritic spines. When a neurotransmitter binds to this type of receptor, the channel opens, allowing the corresponding ions to cross the membrane. Ligand-gated channels that allow positively charged ions (i.e., cations) to enter the cell favor the formation of a new action potential and therefore are excitatory. Ligand-gated channels that allow negatively charged ions (i.e., anions) to enter the cell make it more difficult to induce an action potential and therefore are inhibitory. In general, ionotropic receptors produce relatively fast actions at the synapse that are relatively short lived and therefore mediate rapid behaviors.
- Metabotropic, or second messenger-linked, receptors are located at the synapse but may also be found in the membrane around the synapse (i.e., perisynaptic membranes) and on the transmitting cell’s presynaptic membrane. These receptors are not linked to ion channels but act on ion channels through an indirect mechanism. When the receptor becomes activated by its neurotransmitter, it acts on intermediary molecules (G-proteins) to release a second messenger called cyclic AMP (cAMP). cAMP, in turn, acts on the ion channel to allow ions to move into or out of the neuron. In addition, cAMP helps control numerous other processes in the cell. Metabotropic receptors generally produce slower and longer-lasting reactions at the synapse that have modulatory effects rather than generate new nerve signals.

Each neuron carries receptors for both excitatory and inhibitory neurotransmitters on its surface; moreover, some of the signals will be mediated through ionotropic receptors and induce fast responses whereas others will be mediated through metabotropic receptors and trigger slow responses. The integration of all the incoming, often conflicting, signals determines whether the neuron will generate a new signal (i.e., a new action potential) that can be passed on to other neurons.

*—Peter Clapp, Ph.D.; Sanjiv V. Bhave, PhD and Paula L. Hoffman, Ph.D*.

### Role of Glutamate Systems During Ethanol Withdrawal

As a result of increases in iGluR expression and function induced by chronic ethanol exposure, the central nervous system enters a state of excessive activation (i.e., hyperexcitability) when ethanol is suddenly withdrawn. In animals, this state is characterized by seizure activity. These seizures can be prevented by NMDAR antagonists that either block the receptor channel (e.g., an agent called dizocilpine [MK-801]) or which bind to certain sites on the receptor and thereby interfere with the normal interaction between agonists and the NMDAR (Kotlinska and Liljequist 1996; Veatch and Becker 2005). Withdrawal after chronic ethanol treatment also elicited prolonged and excessive NMDAR-dependent activity in certain neurons (i.e., CA1 pyramidal neurons) isolated from rat hippocampus that is similar to the activity observed during epileptic seizures (Hendricson et al. 2007). The ethanol withdrawal–induced hyperexcitability predisposes neurons to excitotoxic cell death if the NMDARs are stimulated. Compounds that act as NMDAR antagonists, including MK-801 and ifenprodil, can protect the cells against withdrawal-induced neurotoxicity (al Qatari et al. 2001; Hoffman et al. 1995).

Withdrawal from chronic ethanol exposure not only relieves the persistent inhibition of postsynaptic glutamate receptors but also is associated with elevated glutamate levels outside the neurons (i.e., in the synaptic cleft) in the NAc, hippocampus, amygdala, and dorsal striatum (Dahchour and DeWitte 2003; Roberto et al. 2004*b*; Rosetti and Carboni 1995). It is possible that chronic ethanol exposure leads to reduced numbers or reduced activity (i.e., downregulation) of presynaptic Group II and Group III mGluRs that help control neuro-transmitter release; as a result, glutamate release would be less inhibited and glutamate levels in the synapse would increase. This model is supported by findings that the levels of intermediary molecules (i.e., messenger RNA [mRNA]) that are necessary for the production of mGluR3 and mGluR7 proteins were reduced in the hippocampus of ethanol-fed rats (Simonyi et al. 2004). Moreover, it has been demonstrated that Group II mGluR agonists can effectively prevent seizure activity associated with elevated extracellular glutamate (e.g., Smolders et al. 2004). Alternatively, prolonged ethanol exposure may interfere with the normal removal of glutamate from the synapse by reducing the uptake of the neurotransmitter by adjacent cells called astrocytes (Smith 1997).

The combination of increased postsynaptic NMDAR function and elevated glutamate levels in the synapse found after ethanol withdrawal creates a “hyperglutamatergic” state associated with seizure activity and neuronal injury (see figure 2C). This state may contribute to the signs and symptoms of the acute alcohol withdrawal syndrome, including disorientation, agitation, and anxiety. Withdrawal-related anxiety, in turn, significantly contributes to continued alcohol abuse and may be associated with relapse in abstinent alcoholics. (For more information on the role of anxiety in relapse, see the sections on GABA and CRF.)

### Role of Glutamate Systems in Relapse Drinking

Most of the changes in glutamate receptors observed after chronic ethanol exposure are short-lived and therefore are likely related to signs of acute withdrawal (e.g., convulsions or anxiety) (Gulya et al. 1991; Roberto et al. 2006). However, because of the increases in NMDAR activity, the overall electrical signal that is generated in the postsynaptic cell in response to glutamate release also is stronger—in other words, synaptic strength is increased. This increase in synaptic strength may lead to a phenomenon called “metaplasticity,” whereby the system becomes more sensitive to subsequent synaptic plasticity processes (Lau and Zukin 2007). In this way, the apparent short-term effects of chronic ethanol treatment and withdrawal on glutamatergic transmission could lead to longer-term alterations.

Treatment with NMDAR antagonists to prevent excessive receptor activity when ethanol is withheld can reduce both the alcohol deprivation effect (Vengeliene et al. 2008) and cue-induced reinstatement of alcohol-seeking behavior in rats (Backstrom and Hyytia 2004). In alcohol-dependent humans, these antagonists can reduce cue-induced craving for alcohol, possibly because they can produce subjective effects that resemble those produced by alcohol (Krupitsky et al. 2007; Krystal et al. 1998). Similarly, treatment of animals with AMPAR antagonists reduced cue-induced reinstatement of alcohol-seeking behavior as well as the alcohol deprivation effect (Sanchis-Segura et al. 2006). mGluRs also may be important for relapse drinking. Antagonists at mGluRs have demonstrated similar effects, resulting in reduced alcohol deprivation effect and attenuated anxiety and alcohol-seeking behavior in cue-induced reinstatement models of relapse (Backstrom and Hyytia 2005; Backstrom et al. 2004; Busse et al. 2004; Zhao et al. 2006).

The agent acamprosate, which has prolonged abstinence in alcohol-dependent patients in some studies (see Kranzler and Gage 2008) and is approved for the treatment of alcohol dependence in the United States, appears to act on both NMDA and mGluR5 receptors (Spanagel and Kiefer 2008). Thus, acamprosate inhibits NMDAR-mediated calcium influx in cultured rat neurons from some, but not all, brain regions (Allgaier et al. 2000; Popp and Lovinger 2000). Moreover, acamprosate recently was shown to inhibit mGluR5 signaling (Harris et al. 2003) and is ineffective in mice lacking mGluR5 (Blednov and Harris 2008). In general, acamprosate appears to restore the balance between excitatory (i.e., glutamate) and inhibitory (i.e., GABA) neuro-transmission following chronic alcohol consumption and withdrawal (De Witte et al. 2005).

Topiramate, an anticonvulsant medication, is another compound that can attenuate alcohol craving and consumption (Anderson and Oliver 2003; Rubio et al. 2004). It also has multiple mechanisms of action, including inhibition of kainate iGluRs and activation of GABA receptors (Gibbs et al. 2000; White et al. 1997). In recent clinical trials, treatment with topiramate resulted in significant favorable drinking outcomes as well as improved physical and psychosocial well-being of alcohol-dependent patients (Florez et al. 2008; Johnson et al. 2008; Krupitsky et al. 2007).

It still is unclear whether the agents tested so far alter the plasticity changes associated with chronic alcohol consumption and withdrawal. Nevertheless, understanding the alcohol-induced changes in glutamatergic transmission already has helped researchers develop therapeutic approaches for treating alcohol dependence.

## Opiate Systems and Alcohol Dependence

**Figure 3:**
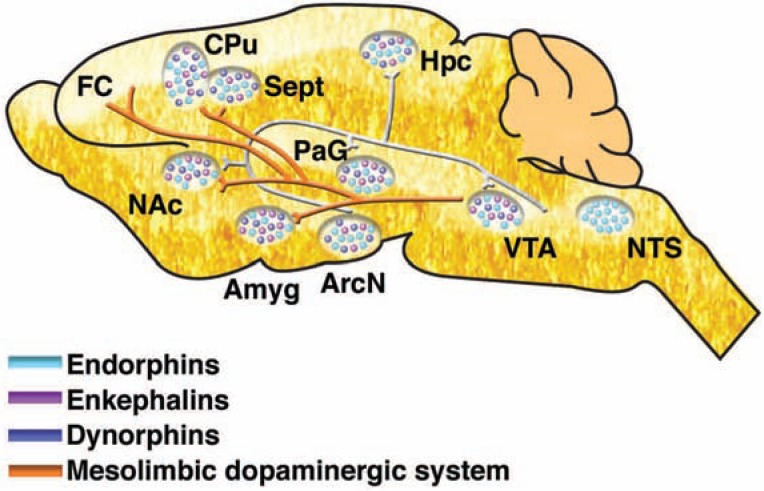
Lengthwise view of the rat brain showing the distribution of opioid peptide–producing neurons. The opioid peptides—endorphins (teal), enkephalins (purple), and dynorphins (blue)—and the neurotransmitter dopamine are involved in the processes of reward and reinforcement. Endorphin-producing neurons are located primarily in the arcuate nucleus (ArcN) of the hypothalamus and the nucleus tractus solitarius (NTS); they extend to and release endorphin in various brain areas (purple). Nerve cells in several regions produce enkephalins and dynorphins, which may be released either in the same region or in distant regions through networks of neurons (not shown). The mesolimbic dopamine system (orange line) is influenced by the actions of endogenous opioids and carries dopamine from the ventral tegmental area (VTA) to various parts of the brain (see also figure 1). NOTE: Amyg = amygdala; CPu = caudate putamen; FC = frontal cortex; Hpc = hippocampus; NAc = nucleus accumbens; PaG = periaqueductal gray area; Sept = septum. SOURCE: Gianoulakis, C. Alcohol-seeking behavior: The roles of the hypothalamic-pituitary-adrenal axis and the endogenous opioid system. *Alcohol Health & Research World* 22(3):202–210, 1998. PMID: 15706797

Endogenous opioids are small molecules naturally produced in the body that have similar effects as opiate drugs, such as morphine and heroin. There are three major classes of endogenous opioid peptides: endorphins, enkephalins, and dynorphins. Each of these types of peptides is formed from larger precursor molecules that, depending on the enzymes present in a particular cell, are cut into smaller opioid molecules which then are released from the cells (Oswald and Wand 2004):

- β-Endorphin is generated from the precursor pro-opiomelanocortin (POMC), which is synthesized in the anterior pituitary (and in the intermediate lobe of the pituitary in rodents), as well as in neurons of the arcuate nucleus of the hypothalamus and in the nucleus tractus solitarius (see figure 3). Fibers containing endorphin project from the arcuate nucleus to other hypothalamic nuclei as well as to the septum, NAc, peri-aqueductal gray area, amygdala, and hippocampus.
- Met- and Leu-enkephalin are produced from the precursors proenkephalin A and B and prodynorphin; neurons that synthesize proenkephalin are widely distributed in brain.
- Dynorphin is generated from prodynorphin. Cells containing dynorphin are found in the hypothalamus, cortex, amygdala, and other brain regions.

To exert their effects, the endogenous opioid peptides interact with three subtypes of receptors (Zöllner and Stein 2007):

- The μ receptor, which has high affinity for β-endorphin and lower affinity for enkephalins;
- The δ receptor, which has high affinity for enkephalins and lower affinity for endorphins; and
- The κ receptor, which is more selective for dynorphins.

Endogenous opioids that interact with μ and δ receptors have positive reinforcing properties. In particular, animals will self-administer β-endor-phin, and the opioid has a high abuse potential, similar to synthetic opiates such as morphine (Van Ree et al. 2000). These and other findings suggested that modification of the endogenous β-endorphin system could play a role in the development of AOD dependence in general.

### Effects of Ethanol Exposure on Opiate Systems

#### Effects on β-Endorphin

Ethanol increases β-endorphin release from the pituitary and hypothalamus in vitro. This effect displays an inverse U-shaped dose-response curve, meaning that lower ethanol concentrations produce a greater effect than higher concentrations (de Waele and Gianoulakis 1993; Gianoulakis 1990). Moreover, in vivo studies found that acute ethanol administration to rodents increased the POMC content of the pituitary, the release of pituitary and hypothalamic β-endorphin, and β-endorphin levels in the blood (Gianoulakis 1993; Modesto-Lowe and Fritz 2005). In some studies (Gianoulakis 1993; Modesto-Lowe and Fritz 2005), the effect of ethanol on β-endorphin was greater in alcohol-preferring than in alcohol-avoiding selectively bred lines of animals.

The effects of chronic ethanol treatment on rodent pituitary and hypothalamic β-endorphins, either in vitro or in vivo, appear to depend on the species and strain of animal tested, the ethanol dose or concentration used, the duration of exposure, and the pattern of alcohol administration in vivo (e.g., intermittent versus constant exposure). Until the influence of these factors has been more clearly defined, it is difficult to determine under which conditions the activity or levels of hypothalamic and pituitary β-endorphin are increased or decreased during and after chronic alcohol exposure (Modesto-Lowe and Fritz 2005; Oswald and Wand 2004). Furthermore, little information is available on potential changes in β-endorphin in other brain regions.

### Effects on Enkephalins and

#### Dynorphins

Ethanol also can affect the levels of proenkephalin- and pro-dynorphin-derived opioids; however, the effects of acute and chronic exposure vary among studies (Modesto-Lowe and Fritz 2005; Oswald and Wand 2004). Similarly, the reported effects of acute and chronic ethanol exposure on brain opioid receptors have varied (Gianoulakis 1993; Oswald and Wand 2004). This variation may result from the fact that ethanol can have different effects on ligand binding to the receptors, depending on its concentration, and can interact with other factors that modulate receptor binding in in vitro tests (e.g., Hoffman et al. 1984; Tabakoff and Hoffman 1983).

Overall, the most consistent effect of alcohol on the opioid systems appears to be an acute increase in β-endorphin release from the pituitary and hypothalamus, with a few reports that alcohol increases endorphin levels in the NAc and VTA (Olive et al. 2001; Rasmussen et al. 1998). The most convincing evidence for a role of the opiate systems in alcohol drinking and dependence, however, comes not from direct analyses of alcohol’s effects on endogenous opioids or opiate receptors, but from behavioral and neurochemical studies using opiate receptor antagonists, such as naloxone and naltrexone.

### Impact of Opioid Antagonists on Alcohol’s Effects on the Brain

As mentioned earlier, alcohol exposure affects numerous neurotransmitter systems, and some of these effects appear to be mediated or moderated by the endogenous opioid system. For example, as described before, acute alcohol exposure increases dopamine release from neurons localized in the VTA, which likely promotes alcohol self-administration and consumption (as well as self-administration of other drugs of abuse) (Di Chiara and Bassareo 2007; Spanagel and Weiss 1999). Some evidence suggests that opiate systems also are involved in this process. For example, when mice were treated with the μ receptor antagonist, naloxoazine, the ethanol-induced dopamine release in the NAc was reduced (Job et al. 2007). The same result was found in animals that were genetically altered so that they no longer produced a functional μ receptor (i.e., when the μ receptor gene was “knocked out”) (Job et al. 2007).

**Figure 4:**
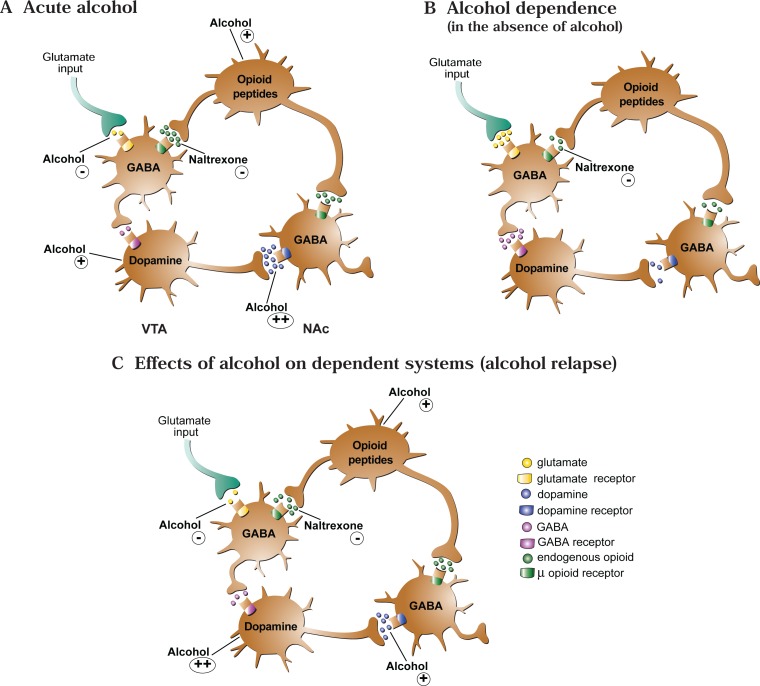
Alcohol’s effects on endogenous opioids and the mesolimbic dopamine system. The activity of the dopamine-releasing (i.e., dopaminergic) neurons in the ventral tegmental area (VTA) is controlled by γ –aminobutyric acid (GABA)-releasing (i.e., GABAergic) neurons. When these GABA neurons are activated (e.g., through the actions of the excitatory neuro-transmitter glutamate), their signals decrease the firing of dopaminergic neurons. Endogenous opioids, however, can act on μ receptors on the GABAergic neurons, thereby inhibiting GABA transmission, and ultimately leading to increased dopamine release. **A)** Acute alcohol can induce β-endorphin release, resulting in activation of μ receptors on the GABAergic neurons in VTA. This, in combination with alcohol’s inhibition of glutamate effects on GABA neurons, could lead to decreased GABAergic activity in the VTA, and subsequently increased firing of the dopaminergic neurons, resulting in increased dopamine release in the nucleus accumbens (NAc). Alcohol also directly increases the activity of dopamine neurons. **B)** During withdrawal from alcohol, after chronic alcohol exposure that produces alcohol dependence (i.e., in the absence of alcohol in a dependent individual), glutamate input to GABA neurons is increased, leading to decreased dopamine release. In addition, the activity of the VTA dopamine neurons is reduced. **C)** When alcohol is reintroduced, the dopamine neurons are more sensitive to alcohol’s direct effects; moreover, alcohol again inhibits glutamate β-endor-phin release, thereby reversing the decreased dopamine release that occurs in the alcohol-abstinent, alcohol-dependent individual. NOTE: Other systems that interact with alcohol to control dopamine neuron activity in the VTA (and dopamine release in the nucleus accumbens), but that are not shown in this figure, include endogenous cannabinoids (which can affect GABA release and interact with opioid systems), nicotinic cholinergic receptors, and serotonin transmission.

The pathway from alcohol exposure to increased dopamine release seems to involve the inhibitory neuro-transmitter GABA as well as opioid systems (Cowen and Lawrence 1999) (For more information on the GABA system, see the following section.) In the VTA, the activity of the dopamine-releasing (i.e., dopaminergic) neurons normally is controlled by GABA-releasing (i.e., GABAergic) neurons. When these GABA neurons are activated, their signals decrease the firing of dopamin-ergic neurons. Endogenous opiates, however, can act on μ receptors on the GABAergic neurons, thereby inhibiting GABA transmission and ultimately leading to increased dopamine release (Di Chiara and North 1992; Margolis et al. 2003). Therefore, it is possible that ethanol can induce β-endorphin release, resulting in activation of μ receptors in the VTA. This, in turn, could lead to decreased GABAergic activity in the VTA and, subsequently, increased firing of the dopaminergic neurons in the VTA (Xiao et al. 2007) (see figure 4).^12^ This hypothesis is supported by many animal studies demonstrating that treatment with naloxone and naltrexone reduced the animal’s alcohol consumption both by affecting the palatability of alcohol and by inducing postingestive changes, such as effects on mesolimbic dopamine release as described here^13^ (Coonfield et al. 2002; Davidson and Amit 1997; Krishnan-Sarin et al. 1998).

### Role of Opioids and Opioid Receptor Antagonists During Alcohol Withdrawal

The dopamine system, which as described above is controlled at least in part by the opioid system, plays an important role in alcohol withdrawal. Studies in which alcohol was withheld for 8 hours from rats that had ingested alcohol in a liquid diet for several weeks suggest that dopamine release in the NAc is reduced during acute alcohol withdrawal but returns to control levels if the animals are allowed to self-administer alcohol (Weiss et al. 1996). This decreased dopamine release during withdrawal may result from a decreased number of spontaneously active dopaminergic neurons in the VTA (Shen 2003). Moreover, additional studies in mice found that not only can alcohol administration return dopamine release to control levels after withdrawal, but dopaminergic neurons in the VTA of alcohol-withdrawn mice actually may be more sensitive to alcohol’s effects (i.e., may show greater ethanol-induced increases in firing rate) (Brodie 2002). In addition, the dopaminergic neurons in the VTA of the alcohol-withdrawn animals exhibited a decreased inhibitory response to GABA, which could contribute to increased dopamine release after ethanol exposure (Brodie 2002). Together, these observations suggest that a type of sensitization to ethanol occurs in the VTA neurons of alcohol-withdrawn mice.

As mentioned before, μ receptor antagonists can reduce the portion of the acute effect of alcohol on dopamine release in the VTA that is mediated through endorphin release. These antagonists still can attenuate alcohol’s enhanced effect on dopamine release after withdrawal, and in this way they could contribute to a reduced alcohol consumption by the withdrawn animals.

Studies found that in some instances, mesolimbic dopamine release in animals is altered for longer periods after alcohol withdrawal (Diana et al. 2003; Thielen et al. 2004). Furthermore, researchers found large decreases in dopamine release in the ventral striatum of detoxified alcohol-dependent humans (Volkow et al. 2007). Such long-term decreases in baseline dopamine release, combined with increased sensitivity to the dopamine-releasing effects of alcohol, could represent a basis for relapse drinking after a period of abstinence. However, as described above, these changes would be sensitive to blocking by opiate receptor antagonists. Indeed, μ receptor antagonists can block cue- and alcohol-induced reinstatement of alcohol consumption in rats (Bienkowski et al. 1999; Lê et al. 1999). Similarly, the efficacy of nal-trexone in reducing excessive drinking in alcohol-dependent people may result from the agent’s ability to reduce reinstatement of alcohol drinking, possibly by interfering with alcohol’s reinforcing effects (e.g., Pettinati et al. 2006). However, individuals differ in the development of sensitization to alcohol’s effect on dopamine release as well as in the nature of changes in other systems (e.g., GABA, glutamate, and serotonin) that modulate these effects. These differences may account for the relatively small overall effect that naltrexone has in reducing excessive drinking by alcohol-dependent people (Donovan et al. 2008).

## GABA Systems and Alcohol Dependence

GABA is the major inhibitory neuro-transmitter in the central nervous system. It acts both on the axon terminal region of presynaptic neurons and on the synaptic region of postsynaptic neurons. In presynaptic neurons, GABA’s actions make it more difficult for the cell to release its normal neurotransmitter, including GABA itself. Thus, in tissue samples obtained from the hippocampus, activation of presynaptic GABA receptors resulted in inhibition of GABA release (Axmacher and Draguhn 2004; Ruiz et al. 2003). In postsynaptic neurons, GABA generally makes it more difficult to generate an electrical signal, thereby interfering with further signal transmission. To exert these effects, GABA acts via presynaptic and postsynaptic ionotropic (GABA_A_) and metabotropic (GABA_B_) receptors. The GABA_A_ receptor, which is expressed widely in the central nervous system, is a protein complex that is linked to a chloride channel. When activated by GABA, the channel opens to allow chloride ions to pass through the cell membrane, thereby increasing the difference in electrical charge between the inside and outside the cell (Mohler 2006; Sieghart and Sperk 2002) (see figure 5A). Through this mechanism, GABA_A_ receptor-coupled chloride channels mediate fast synaptic inhibition in the brain. GABA_B_ receptors, in contrast, like mGluRs, are linked to G-proteins (see Bettler and Tiao 2006; Kornau 2006).

### GABA_A_ Receptors

GABA_A_ receptors have been implicated in a variety of conditions, including stress, anxiety, depression, epilepsy, insomnia, and learning and memory; in addition, they contribute to various acute effects of alcohol, such as sedation and anxiolysis (Johnston 2005; Mohler 2006; Sieghart and Sperk 2002).The action of GABA on GABA_A_ receptors is further enhanced by sedative agents, such as benzodiazepines, barbiturates, and general anesthetics, which do not bind to the same site on the receptor as GABA but act at different sites.

Each GABA_A_ receptor is made up of five subunits. Many different classes of receptor subunits—known as α, β, γ, δ, ɛ, θ, and π subunits—have been identified, and for some classes there is more than one type of subunit (e.g., α1 to α6, and β1 to β3). The specific composition of a given receptor molecule determines its distinct physiological and pharmacological properties. The different subunits also are produced in different regions of the animal and human nervous system (i.e., have distinct expression patterns) (see Michels and Moss 2007; Sieghart and Sperk 2002) and are located in different regions of the neuron (e.g., presynaptically, in the synaptic region of the postsynaptic cell, or in the membrane more distant from the synapse [i.e., in the extrasynaptic region]) (Michels and Moss 2007). For example, whereas synaptic GABA_A_ receptors contain α1, α3, or α5 subunits as well as γ1 or γ2 subunits, extrasynaptic GABA_A_ receptors contain α4, α6, and δ subunits. The subunit composition also affects the affinity of the receptors for their ligands. The synaptic GABA_A_ receptors have relatively low affinity for GABA compared with extrasynaptic receptors; conversely, extrasynaptic receptors are relatively insensitive to benzodiazepines. Moreover, activation of synaptic and extrasynaptic GABA_A_ receptors leads to inhibitory effects through different mechanisms (Michels and Moss 2007). Activation of synaptic GABA_A_ receptors is dependent on GABA release at the synapse and may result in a short-term inhibitory effect (known as phasic inhibition). Activation of extrasy-naptic GABA_A_ receptors plays a role in producing a stable electrical current that is present in neurons at their resting potential and is not dependent on synaptic GABA release (known as tonic inhibition).

Several proteins associate with the GABA_A_ receptor subunits and modulate GABA_A_ receptor function by influencing receptor trafficking, stabilizing the receptors, or modifying the receptors by posttranslational modification as described below (Chen and Olsen 2007; Coyle and Nikolov 2003). As with the glutamate receptors described above, recent studies (Chen and Olsen 2007; Michels and Moss 2007) have suggested that redistribution of GABA_A_ receptors may play a role in synaptic plasticity. Such receptor trafficking can involve movement of the receptor from synaptic to extrasynaptic regions of the cell as well as uptake of receptor molecules into the cell (Bogdanov et al. 2006).

In addition to subunit composition and association with other proteins, posttranslational modification also influences the exact function of specific GABA_A_ receptor molecules. These modifications occur after the proteins comprising the receptor have been synthesized. At that point, other enzymes perform modifications, such as addition of phosphate groups (i.e., phosphorylation), that can influence receptor function (Brandon et al. 2002; Sigel 1995) and trafficking (Kittler and Moss 2003). For example, most GABA_A_ receptor subunits have sites where phosphorylation can occur (Macdonald and Olsen 1994), and phosphorylation of GABA_A_ receptor subunits by different kinases (e.g., PKA and PKC) has been observed. Phosphorylation also is important for the effects of modulators such as benzodiazepines on GABA_A_ receptor function (see Kittler and Moss 2003).

### Effects of Acute Alcohol Exposure on the GABA System

Alcohol has sedative and anxiety-reducing (i.e., anxiolytic) effects, similar to those of barbiturates and benzodiazepines, which are known to act at the GABA_A_ receptor. Consequently, many studies have investigated the interactions of alcohol with GABA_A_ receptors. In general, these studies found that acute alcohol exposure enhances GABAergic neurotransmission (see figure 5B). However, the mechanism(s) by which this effect occurs, and the adaptations in the systems after chronic alcohol exposure and withdrawal, still are being discovered (see Grobin et al. 1998; Wallner et al. 2006).

The hypothesis that the GABA system helps mediate alcohol’s acute effects was supported by early studies demonstrating that several behavioral effects of acute alcohol exposure were enhanced by GABA_A_ receptor agonists and attenuated by antagonists. For example, benzodiazepines, which are positive modulators of GABA_A_ receptor function, potentiated ethanol’s anxiolytic effects (Ho and Yu 1991). Conversely, different GABA_A_ receptor antagonists decreased ethanol-induced intoxication (i.e., ataxia) (Martz et al. 1983; Suzdak et al. 1986) and sedation (Givens and Breese 1990). These and other findings suggested that alcohol exerts some of its acute effects by enhancing GABAergic neurotransmission (see Grobin et al. 1998; Wallner et al. 2006).

Additional in vitro studies demonstrated that low concentrations of ethanol potentiate GABA_A_ receptor function in different experimental systems (Allan and Harris 1986; Suzdak et al. 1986; Ticku and Burch 1980). However, electrophysiological analyses of ethanol’s effects on GABA_A_ receptor function did not yield consistent results (Grobin et al. 1998; Wallner et al. 2006). In some cases, very specific conditions (e.g., a specific test temperature) were needed to observe any effects, which raised questions regarding the physiological significance of these effects. At least in part, the variability of ethanol’s effects results from differences in the subunit composition of the GABA_A_ receptors in different cells. For example, receptors that contain the δ subunit may be most sensitive to ethanol-induced increases in activity (Sundstrom-Poromaa et al. 2002). Because receptors with this subunit typically are extrasynaptic, this would suggest that ethanol has greater effects on tonic inhibition than on phasic inhibition by synaptic GABA_A_ receptors. Other investigators, however, have questioned whether the presence of the δ subunits does, in fact, lead to more potent effects of ethanol (Borghese et al. 2006; Korpi et al. 2007; Krystal et al. 2006; Mody 2008; Sundstrom-Poromaa et al. 2002).

How exactly ethanol affects GABA_A_ receptor function is unclear. Although some researchers have proposed that ethanol binds directly to GABA_A_ receptors (Wick et al. 1998), the variability of results suggests that alcohol affects receptor function more indirectly (e.g., via phosphorylation events). This hypothesis is supported by observations that when phosphorylation is prevented by inhibiting PKC, the receptors’ sensitivity to ethanol is reduced (Weiner et al. 1994). Similarly, some studies found that receptors obtained from mice that lack a certain PKC variant were less sensitive to ethanol than receptors from normal mice (Bowers et al. 1999; Harris et al. 1995; Weiner et al. 1994). However, receptors from mice that lack another PKC variant, or from mice in which that PKC variant is inhibited, showed increased sensitivity to ethanol and benzodiazepine potentiation (Hodge et al. 1999; Proctor et al. 2003; Qi et al. 2007). Thus, the exact role that phosphorylation by PKC plays in mediating ethanol’s effects on the GABA_A_ receptor depends on the presence or absence of particular forms of the kinase in a given cell. Moreover, PKA also appears to influence ethanol’s effect on GABA_A_ receptor function, at least in some cell types (Freund and Palmer 1997; Wang et al. 1999).

Another intriguing possibility for how ethanol can indirectly affect GABA_A_ receptor function involves neuroactive steroids—steroid molecules that are naturally produced in the adrenal glands, ovaries, testes, and brain and which can act on GABA_A_ receptors and modulate their function. For example, these steroids can enhance GABA_A_ receptor function, which leads to anxiolytic, pain-reducing (i.e., analgesic), and anticonvulsant effects (see Girdler and Klatzkin 2007; Mitchell et al. 2008). Extrasynaptic GABA_A_ receptors that contain the δ subunit seem to be particularly sensitive to the effects of the steroids (Mody 2008; Morrow 2007). The hypothesis that ethanol’s actions involve neuroactive steroids stems from the observation that systemic ethanol administration at relatively low doses increases plasma and brain levels of certain neuroactive steroids; moreover, ethanol can increase synthesis of these steroids in brain (see Biggio et al. 2007). Numerous studies have provided evidence that this elevation in neuroactive steroid levels may contribute to various behavioral effects of ethanol by modulating GABA_A_ receptor function (see Morrow 2007) (figure 5B).

Ethanol may not only modulate the function of GABA_A_ receptors directly or indirectly but also may act presynaptically to increase GABA release in numerous brain regions (Ariwodola and Weiner 2004; Nie et al. 2004). In the amygdala, the effect of ethanol on GABA release appears to be mediated by activation of CRF receptors (Nie et al. 2004), and other reports suggest a similar role for an opiate-like receptor (i.e., the noci-ceptin receptor) (The CRF system is discussed in the section “Stress, CRF, and Alcohol Dependence”).

In one brain region, however, ethanol decreases rather than increases GABAergic neurotransmission—in the VTA (Stobbs et al. 2004; Xiao et al. 2007). As mentioned earlier, this area contains cell bodies of neurons that release dopamine into the NAc. The dopamine neurons in the VTA are continuously inhibited (i.e., are under tonic inhibitory control) by GABA-containing neurons (Johnson and North 1992); accordingly, an ethanol-induced decrease in GABAergic neurotransmission leads to increased mesolimbic dopamine release. Ethanol appears to decrease GABAergic transmission in part by inhibiting NMDA receptors that normally serve to increase GABA release in response to signals mediated by glutamate (Steffensen et al. 1998). In addition, ethanol appears to reduce GABA transmission by activating certain receptors from the opioid system.

### The GABA System and Alcohol Consumption

As noted earlier, ethanol-mediated potentiation of GABA function is thought to contribute to the acute anxiolytic and sedative effects of ethanol. More direct evidence indicates that the GABA system helps modulate alcohol consumption. For example, treatment of animals with GABA_A_ receptor antagonists generally decreases alcohol self-administration (Rassnick et al. 1993; Samson et al. 1987). Conversely, treatment with a neuroactive steroid that enhances GABA_A_ receptor function increased alcohol intake (Janak and Gill 2003). Together, these findings suggest that ethanol-mediated enhancement of GABA_A_ receptor function or GABA release (which would produce an anxiolytic effect) promotes alcohol consumption (Koob 2004). One interpretation of the results is that if one blocks the effect of alcohol by treating the animal with a GABA_A_ receptor antagonist, the animal does not feel the anxiolytic effect of alcohol and reduces its alcohol consumption. However, the results of such experiments are difficult to interpret because ethanol intake also can be decreased if a compound substitutes for ethanol rather than blocks ethanol’s effect, and the animal therefore no longer “needs” alcohol. For example, if ethanol decreases GABA function in critical VTA neurons, thereby increasing dopamine release, treatment with a GABA receptor antagonist would not block the effect of ethanol but instead might have the same effect as ethanol on dopamine release. Therefore, the animal treated with the antagonist would no longer need to consume ethanol to experience this effect. Furthermore, when the agonists or antagonists are administered not directly into the brain but in other areas of the body (e.g., with the food or by injections) as in these studies, it is not possible to determine the specific neuronal pathways that are being affected. However, direct injection of a GABA_A_ receptor antagonist into the extended amygdala—which includes the amygdala itself as well as the brain regions that send projections to, or receive projections from, the amygdala, such as the NAc—also reduces alcohol intake (Hyytia and Koob 1995; June et al. 2003). Because alcohol appears to enhance GABA neu-rotransmission in these brain regions (Hodge and Cox 1998; Nie et al. 2004), the interpretation is that the GABA_A_ receptor antagonist is blocking the effect of alcohol.

Agonists acting at GABA_B_ receptors also reduce alcohol intake in selectively bred alcohol-preferring rats (Maccioni et al. 2008; Quintanilla et al. 2008) and in rats trained to press a lever to receive alcohol (Janak and Gill 2003). GABA_B_ receptors are located presy-naptically, where they can inhibit GABA release, and postsynaptically, where they mediate neuronal inhibition (Cryan and Kaupmann 2005). There is evidence that activation of GABA_B_ receptors—whether by agonists or by ethanol—can reduce anxiety (Cryan and Kaupmann 2005). Accordingly, treatment with a GABA_B_ agonist could substitute for the anxiolytic effect of ethanol, leading to its reduced consumption. One would expect that treatment of animals with a GABA_B_ receptor antagonist might also reduce ethanol intake, but in this case, it would be because the animal would not feel the anxiolytic effect of ethanol.

Other evidence for a role of GABA systems in alcohol consumption comes from studies of mice lacking different variants of PKC. Mice that lacked one type of PKC, and in which GABA_A_ receptor function was less sensitive to potentiation by ethanol, demonstrated increased ethanol self-administration compared with normal mice (Harris et al. 1995). Conversely, mice that lacked another type of PKC, and in which GABA_A_ receptor function was more sensitive to potentiation by ethanol, consumed less ethanol (Song and Messing 2005). These animals appear to be more sensitive to ethanol’s aversive effects and less sensitive to its rewarding effects (Newton and Messing 2007). Together these findings suggest that potentiation of GABA transmission by ethanol modulates the animals’ motivation to consume ethanol.

### Effects of Chronic Alcohol Exposure on the GABA System

The acute effects of ethanol on pre- and postsynaptic GABA signaling described above suggest that GABAergic neurotransmission would be decreased following chronic ethanol exposure as an adaptation to persistent activation by ethanol (see figure 5C). This decreased inhibitory activity could contribute to the anxiety and neuronal hyperexcitability observed during acute alcohol withdrawal. Indeed, in early studies GABA_A_ receptor agonists exhibited decreased biochemical effects in certain brain regions of chronically ethanol-treated animals (Morrow et al. 1988) or after chronic in vitro exposure of cells to ethanol (Buck and Harris 1991). In contrast, other studies found no change in the response to GABA_A_ agonists (Allan and Harris 1987; Tremwel et al. 1994), and studies of ligand binding to GABA_A_ receptors also did not reveal consistent reductions in receptor numbers (see Tabakoff and Hoffman 1996). Furthermore, electrophysiological analyses of brain samples from ethanol-withdrawn animals suggested that the observed seizures did not arise from changes in GABA_A_ receptor function (Ripley et al. 1996). A more recent study (Olsen et al. 2005) using chronic intermittent alcohol exposure (i.e., several episodes of ethanol exposure and withdrawal), however, reported impaired GABA_A_ receptor function in the hippocampus; moreover, the animals exhibited greater susceptibility to seizures and increased anxiety. Reduced activity of GABA_A_ receptors could contribute to the efficacy of benzodiazepines, which potentiate the activity of many subtypes of GABA_A_ receptors, in controlling seizures and convulsions induced by alcohol withdrawal. These drugs commonly are used to treat acute symptoms of alcohol withdrawal (Schuckit and Tapert 2004).

Interestingly, chronic ethanol administration has the opposite effect on the activity of GABA neurons in the VTA as on GABA systems in other brain areas—that is, the VTA neurons show increased activity (Gallegos et al. 1999). This increase may reflect the increased glutamater-gic activity that occurs during alcohol withdrawal and which was described earlier. This increased GABA activity would contribute to the decreased mesolimbic dopamine release associated with withdrawal (see figure 4).

The difficulty in demonstrating consistent changes in GABA_A_ receptor function in dependent animals results, at least in part, from the complex changes in the production of different GABA_A_ receptor subunits induced by chronic alcohol administration and withdrawal. These changes depend on the treatment regimen, the time after withdrawal at which measurements are taken, and the brain area examined (Cagetti et al. 2003). The most consistent effects appear to be a decrease in the production of α1 subunits and an increase in the production of α4 subunits (see Biggio et al. 2007; Follesa et al. 2006; Krystal et al. 2006; Kumar et al. 2004; Olsen et al. 2005). For the δ subunit, in contrast, the findings varied. Thus, one study (Follesa et al. 2006) reported that production of this subunit after alcohol withdrawal was decreased in cells from the cerebellum and increased in neurons from the hippocampus. In contrast, a study using chronic intermittent alcohol exposure found that production of the δ subunit was decreased in the hippocampus (Olsen et al. 2005). Despite these inconsistencies, it appears that chronic alcohol exposure and withdrawal can alter the subunit composition of some GABA_A_ receptors.

Chronic alcohol treatment also may alter the localization of GABA_A_ receptors, similar to the findings with glutamate receptors (see figure 5C). The changes in subunit composition could contribute to this redistribution, because certain subunits are clustered in the synapse as they interact with receptor-associated scaffolding proteins (Krystal et al. 2006). Altered localization and/or subunit composition also influence the sensitivity of GABA_A_ receptors to alcohol, benzodiazepines, and neuroactive steroids as well as the characteristics of tonic and phasic inhibitory neurotransmis-sion, as follows:

- Receptor sensitivity to neuroactive steroids may be decreased or increased, depending on whether more or less of the δ subunit is produced ^14^ (Follesa et al. 2006; Olsen et al. 2005).
- Increases in the α4 subunit decrease sensitivity to the benzodiazepine diazepam and may contribute to the ability of the medication flumazenil to reduce anxiety associated with alcohol withdrawal (Knapp et al. 2004). Flumazenil normally acts as a benzodiazepine receptor antagonist but has the properties of benzodiazepine agonist at receptors containing the α4 subunit (Wafford et al. 1996).
- The decrease in the α1 subunit may contribute to tolerance to alcohol’s effect on the GABA_A_ receptor (see Tabakoff and Hoffman 2002) because receptors containing this subunit are sensitive to alcohol potentiation.

Some changes in GABA_A_ receptor function are reversed relatively rapidly after alcohol withdrawal (e.g., Petrie et al. 2001) and therefore likely contribute to the anxiety and seizure activity associated with acute withdrawal. Other changes, however, persist for weeks to months after withdrawal (Kang et al. 1996) and could contribute to aspects of dependence such as relapse drinking related to persistent anxiety.

If GABA systems play an important, albeit complex, role in alcohol consumption and alcohol withdrawal, agents that modulate these systems might be useful in the treatment of alcohol dependence. In animal studies, some GABA_A_ receptor antagonists were found to reduce alcohol self-administration in nondependent animals, as described above. Conversely, a GABA_A_ receptor agonist that was injected into the amygdala reduced the enhanced alcohol self-administration seen in dependent animals but did not affect alcohol self-administration by nondependent animals (Roberts et al. 1996). This observation suggests that a change in GABA_A_ receptor function, or in brain circuits involving this receptor, occurs in the dependent animals. In another study (Hodge et al. 1995), administration of a GABA_A_ receptor agonist into the NAc led to early termination of alcohol self-administration, whereas an antagonist also reduced ethanol self-administration but by a different mechanism. Therefore, it is possible that termination of alcohol self-administration is most impaired in the alcohol-dependent animals and can be restored by the GABA_A_ receptor agonist. Alternatively, the GABA_A_ receptor agonist may reduce the enhanced anxiety in the alcohol-withdrawn animals, thereby substituting for alcohol’s anxiolytic effect, so that alcohol is no longer “needed” by the animals.

The GABA_B_ agonist, baclofen, also can reduce alcohol consumption in dependent rats and block cue-induced reinstatement of alcohol-seeking behavior in alcohol-preferring rats (Maccioni et al. 2008; Walker and Koob 2007). Together, these findings implicate GABA systems in aspects of relapse drinking in dependent animals but again suggest that the complexity of adaptations in the GABA receptors is not yet fully understood. Nevertheless, it is important to note that several human studies have now shown evidence of association between alcohol dependence or related characteristics and specific variants in genes coding for GABA_A_ receptor subunits (Dick et al. 2006; Enoch 2008; Matthews et al. 2007).

## Stress, CRF, and Alcohol Dependence

One of the reasons why abstinent alcohol-dependent people relapse may be a long-lasting heightened level of anxiety and/or increased susceptibility to stress following alcohol withdrawal (Breese et al. 2005; Sinha 2007). Alcohol-induced adaptations in GABA and glutamate systems described earlier represent possible mechanisms that sensitize a person to anxiety or stress. Interactions of ethanol with CRF and its receptors (known as CRF1 and CRF2) also may be involved in promoting relapse in alcohol-dependent people by increasing withdrawal-associated anxiety (Heilig and Koob 2007; Koob 2008).

CRF originally was identified as a small protein (i.e., peptide) produced in the hypothalamus that controls the release of adrenocorticotropic hormone (ACTH) from the pituitary gland, which in turn regulates the release of stress hormones (i.e., glucocorticoids) from the adrenal glands. Thus, CRF is a key player in a hormone system known as the hypothalamic–pituitary–adrenal (HPA) axis that is activated under stressful conditions (Herman and Cullinan 1997; Swanson et al. 1986). Acute alcohol exposure can activate this axis, and recent studies (Lee et al. 2004; Li et al. 2005) suggest that alcohol’s effect on the HPA axis requires, among other factors, the presence of CRF in the hypothalamus. However, CRF is produced not only in the hypothalamus but also is found in other brain areas (Cummings et al. 1983). The CRF produced in those areas is thought to play a role in the behavioral stress response (as opposed to the endocrine stress response characterized by the release of stress hormones from the adrenal glands that results from the actions of hypothalamic CRF and pituitary ACTH). The action of CRF is mediated through G-protein–coupled CRF1 receptors in the pituitary and through CRF1 and CRF2 receptors in brain areas such as the extended amygdala (Dautzenberg and Hauger 2002).

A recent review (Heilig and Koob 2007) has well summarized the evidence for a role of CRF and CRF1 receptors in mediating stress/anxiety-induced relapse in alcohol-dependent people, including the following:

- Withdrawal from alcohol after chronic exposure is associated with increased anxiety in animals. This anxiety, which can be observed even long after withdrawal if the animal is subjected to stress, can be blocked by CRF antagonists.
- In animal models in which alcohol consumption is increased following the induction of alcohol dependence (e.g., models of the alcohol deprivation effect, CRF1 receptor antagonists can prevent the increase in consumption; however, these agents do not affect baseline alcohol consumption in nondependent animals.
- CRF1 receptor antagonists block stress-induced reinstatement of alcohol self-administration.
- Both CRF release and the levels of CRF1 receptors are increased in the amygdala of alcohol-dependent/ withdrawn animals. Moreover, these alcohol-withdrawn animals display increased sensitivity to stress and increased alcohol consumption for up to 3 months after withdrawal.

Another study (Lowery et al. 2008) recently found that a CRF1 receptor antagonist also can reduce stress-induced increases in alcohol consumption by nondependent mice. Genetic factors may contribute to the link between CRF, CRF1 receptor, stress sensitivity, and alcohol consumption because selected lines of rats that prefer alcohol and are highly sensitive to stress (msP rats) also have higher levels of mRNA for the CRF1 receptor in the amygdala, apparently because of a variation in the gene that encodes the receptor. When these rats drink alcohol, the production of the CRF1 receptor is decreased (Hansson et al. 2007). Moreover, stress-induced reinstatement of alcohol drinking in the msP rats can be reduced by treatment with a CRF1 receptor antagonist (Hansson et al. 2006). Similarly, researchers found that a specific variant in the CRFR1 gene was associated with high alcohol intake in humans (Treutlein et al. 2006).

In contrast to the CRF1 receptor, production of the CRF2 receptor (as determined by measuring mRNA levels) is decreased in the amygdala of alcohol-dependent animals. Moreover, activation of the CRF2 receptor resulted in decreased alcohol self-administration in dependent animals (Funk and Koob 2007; Sommer et al. 2008).

The molecular mechanism(s) by which increases in CRF and CRF1 receptors in alcohol-dependent animals contribute to anxiety and increased alcohol consumption have not yet been elucidated, but studies have implicated the GABA system in this process. One study found that acute alcohol exposure can increase the release of GABA in the amygdala and that this effect can be blocked with a CRF1 receptor antagonist (Nie et al. 1994). Similarly, CRF itself can promote GABA release in the amygdala via the CRF1 receptor (Bagosi et al. 2008). These effects of ethanol and CRF are not observed in mice lacking a specific variant of PKC (Bajo et al. 2008), suggesting that this enzyme helps mediate the effect of CRF on GABA release. GABA release also is increased in the amygdala of alcohol-dependent rats, possibly because these animals have increased CRF1 receptors; the effect of acute ethanol administration on GABA release in this brain region is unchanged in the dependent animals (Roberto et al. 2004*a*).

Although both CRF and ethanol induce similar changes in GABA release in amygdala, CRF has anxiety-inducing (i.e., anxiogenic) effects, whereas ethanol generally has anxiolytic effects. One explanation for this apparent contradiction could be that the overall anxiolytic effect of ethanol also reflects ethanol-induced enhancement of GABA signaling in regions that receive neuronal projections from neurons in the amygdala (see Bajo et al. 2008). Furthermore, as described in previous sections, ethanol acts not only on the GABA system but also on other neurotransmitter systems. For example, acute ethanol inhibits the activity of postsynaptic glutamate receptors in the amygdala (Roberto et al. 2004*b*), which can have anxiolytic effects (e.g., Kapus et al. 2008; Lack et al. 2007). In addition, chronic alcohol exposure and withdrawal alter pre- and postsynaptic glutamatergic transmission in the amygdala (Lack et al. 2007; Roberto et al. 2004*b*). Further analysis of the interaction of CRF and glutamate in the amygdala of alcohol-naive and alcohol-dependent animals therefore is warranted to better understand the basis for the opposite effects of ethanol and CRF on anxiety levels.

Overall, the studies of CRF suggest that the development of alcohol dependence, particularly after repeated cycles of alcohol exposure and withdrawal, is associated with increased anxiety and increased sensitivity to stress in animals. These changes, which appear to be long-lasting, result, at least in part, from adaptations in the CRF system (i.e., increased CRF release and CRF1 receptors in the amygdala) that contribute to increased alcohol consumption. (The role of changes in other systems that mediate emotional stress, including decreases in the activity of “anti-stress” systems, are detailed in an excellent recent review by Koob [2008].) The changes in CRF (and other systems) in the amygdala are theorized to cause a shift in the motivation for alcohol consumption. Thus, alcohol initially is ingested for its positive reinforcing properties. Once dependence develops, however, a new motivation arises—that is, reduction of the anxiety or stress associated with withdrawal and prolonged abstinence from alcohol, which can be attributed (in part) to increased activity of the brain CRF system (Heilig and Koob 2007; Koob 2008).

## Summary and Conclusions

The adaptations in systems whose activity is modified by acute alcohol exposure and/or that modulate initial alcohol consumption appear to play key roles in the development of alcohol dependence. Both environmental and genetic variables influence a person’s initial alcohol consumption as well as the adaptive changes that occur after chronic alcohol exposure. It is likely that different adaptive responses occur in each person, contributing to some or all of the behaviors associated with alcohol dependence. Because of the variability in adaptive changes, no one therapeutic agent is likely to be effective in all alcohol-dependent people, consistent with the findings of clinical trials (Spanagel and Kiefer 2008).

To investigate and discuss the neurobiology of alcohol dependence, researchers must rely primarily on a range of animal models, including models of acute withdrawal, the alcohol deprivation effect, and reinstatement of alcohol-seeking behavior. Although alcohol dependence as defined by the DSM–IV criteria is not always associated with physiological withdrawal symptoms, they are studied in animal models. Such models of acute withdrawal often rely on the observation of withdrawal seizures and convulsions, which indicate neuronal hyperexcitability. Although these manifestations of withdrawal can be severe (e.g., Ritzmann and Tabakoff 1976), their time course is relatively short in both animals and humans (Gallant 1999; Ritzmann and Tabakoff 1976). The transient increases in glutamate release and glutamate receptor function, and the decreases in GABAergic function, that have been observed when animals were withdrawn from chronic alcohol consumption, likely are central factors in this withdrawal hyperexcitability. In addition, increased activity of certain calcium channels may contribute to withdrawal convulsions (Katsura et al. 2005; Watson and Little 1999; Whittington and Little 1991).

The alcohol deprivation effect, withdrawal-induced alcohol drinking, and reinstatement of alcohol-seeking behavior can be considered to be animal models of other aspects of alcohol dependence in humans as defined by DSM–IV (e.g., relapse drinking or spending time obtaining and drinking alcohol). Accordingly, evidence gained from investigation of these animal models allows researchers to speculate as to the neurobiological basis of alcohol dependence. A construct that may be useful in integrating the data obtained from these models and providing a framework to understand how changes in various neurotransmitter systems contribute to alcohol dependence, proposes that craving for alcohol can arise from different neurobiological sources (Addolorato et al. 2005; Verheul et al. 1999). For example, in some people alcohol consumption would be motivated by craving for reward; this craving could result from changes in the opiate and/or dopamine systems that lead to a reduction of the reinforcing effects of alcohol. As discussed above, dopamine release in the VTA declines during acute withdrawal after chronic alcohol exposure, resulting at least in part from increased glutamatergic activity that in turn leads to increased activity of GABA systems. Moreover, the number of spontaneously active VTA dopamine neurons is lowered during alcohol withdrawal. These baseline changes may contribute to the negative emotional state (i.e., negative affect) that is associated with acute alcohol withdrawal. If, as has been reported, the sensitivity of VTA neurons to direct stimulation by alcohol is increased at the same time, one can conclude that alcohol would be ingested for its rewarding properties. This effect of alcohol could be attenuated by the opiate receptor antagonist naloxone, because μ opiate receptors mediate some of alcohol’s ability to stimulate dopamine release. Although the decreased dopamine release occurs mainly during acute withdrawal, there also is evidence for longer-term reductions in mesolimbic dopamine content or release. These long-term effects might explain why μ opiate receptor antagonists, such as naltrexone, attenuate alcohol-induced reinstatement behavior in animals as well as alcohol intake by alcohol-dependent humans.

A second proposed category of craving is craving for relief from stress or anxiety. A recent review by Koob (2008) focuses particularly on the brain’s stress and “anti-stress” systems that may not only contribute to some degree to the negative-affective state associated with acute alcohol withdrawal but also to the sensitization to stress during protracted abstinence from AODs. As discussed in this review, adaptations in the brain’s CRF systems may contribute to increased anxiety and emotional stress that foster increased alcohol consumption. Consequently, in this situation the motivation for alcohol consumption becomes a quest to reduce anxiety or stress. Additional changes also occur in other brain neuropeptide systems, including increased activity of systems associated with stress and reduced activity of anxiolytic or “anti-stress” systems (Koob 2008). The combination of all of these changes can contribute to stress-induced reinstatement of alcohol consumption. This assumption is supported by the anatomical localization of the observed neurobiological changes. Thus, the changes in brain stress systems have a particular impact in the extended amygdala, which also is influenced by the changes in the dopamine system described above.

Reinstatement of alcohol drinking can be induced not only by stress but also by environmental cues associated with alcohol and by injection of alcohol itself. By using antagonists of various neurotransmitter systems, researchers have been able to investigate which systems are involved in relapse drinking induced by the different stimuli. Such studies found that cue-induced reinstatement, as well as the alcohol deprivation effect, are attenuated by antagonists of both iGluRs and mGluRs. Excessive glutamate activity clearly has been associated with acute withdrawal signs. However, glutamate systems, especially in the hippocampus, also play crucial roles in the synaptic plasticity necessary for learning and memory (Rao and Finkbeiner 2007; Robbins and Murphy 2006). It has been postulated that transient increases in NMDA receptors, such as those seen following acute alcohol withdrawal, can lead to metaplasticity, which is a phenomenon whereby previous synaptic activity can enhance the susceptibility to subsequent synaptic plasticity (Abraham and Bear 1996). Accordingly, long-term alterations in glutamatergic transmission that persist during protracted abstinence may promote a “memory” of alcohol-related cues, leading to cue-induced and alcohol-induced reinstatement of drinking. This process may represent an important target for the glutamate antagonist acamprosate as a therapy to reduce alcohol consumption by dependent humans.

A third proposed category of craving, referred to as obsessive craving (Verheul et al. 1999), is defined as the loss of control over thoughts about alcohol consumption, which intrude into a person’s normal thinking patterns. This type of craving was suggested to result from deficits in serotonin systems (Addolorato et al. 2005). Obsessive craving, including loss of control and compulsive alcohol drinking, however, also could reflect enduring plastic changes in the glutamatergic circuits of the limbic and motor systems as described above (see Kalivas and O’Brien 2008). As a result of these changes, a behavior could become habitual or automatic. As discussed above, it is important to take into account the anatomical localization of the adaptive changes in neurochemical systems. Because the limbic and motor systems control habitual behavior and locomotor activity, changes that impact these systems may be likely to result in automatic activity. On the other hand, changes in serotonin transmission in the cortex, thalamus, and hypothalamus may be associated with obsessive thinking patterns and compulsive drinking.

Although this review has focused on alcohol-induced changes in isolated neurochemical systems, there undoubtedly are interactions between and among these systems that are affected by neuroadaptive changes. For example, recruitment of CRF activity as well as glutamatergic activity in the amygdala of alcohol-dependent animals may generate anxiety. And even if these systems do not interact directly, additive effects can occur that may enhance an individual’s motivation to consume alcohol. Thus, adaptive changes in these and other systems, in particular anatomical regions of brain, can act together, through neurochemical and anatomical connections, leading to the overall syndrome of alcohol dependence. As our understanding grows of the nature of the (mal)adaptive neurobiological changes that occur in each dependent person, the overall goal will be to develop therapies that are tailored to the specific vulnerabilities to neuroadaptation in a particular person and which will therefore provide the needed intervention to prevent or reduce relapse to alcohol drinking.

## Figures and Tables

**Figure 1:**
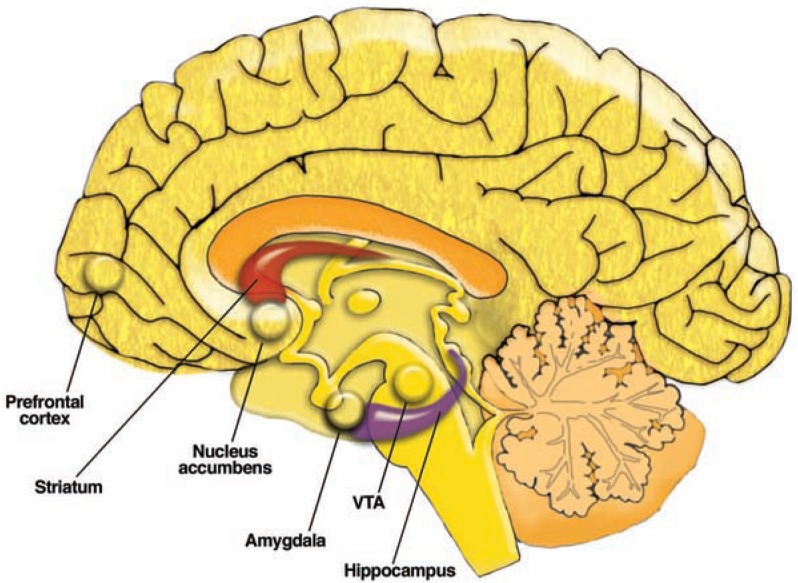
Location of some of the regions in the human brain that are affected by alcohol, including the mesolimbic dopamine system (which includes the ventral tegmental area [VTA], nucleus accumbens, and prefrontal cortex), amygdala, striatum, and hippocampus.

**Figure 2A:**
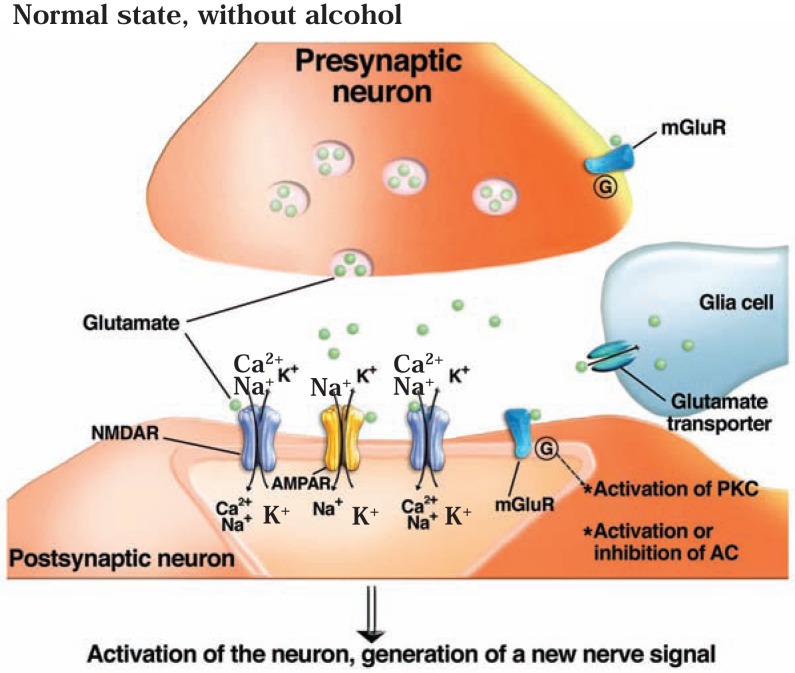
Actions of the brain’s glutamate system. Glutamate (green circles) exerts its effects by acting on various types of receptors, including the *N*-methyl-d-aspartate receptors (NMDARs) and α-amino-3-hydroxy-5-methylisoxazole-4-proprionic acid receptors (AMPARs), both of which are ion channels, and metabotropic glutamate receptors (mGluRs), which are coupled to G-proteins. G-proteins, in turn, indirectly activate protein kinase C (PKC) and activate or inhibit adenyl cyclase (AC), depending on the mGluR and G-protein involved. In the absence of alcohol, glutamate leads to the activation of the postsynaptic neuron and the generation of a new nerve signal.

**Figure 2B:**
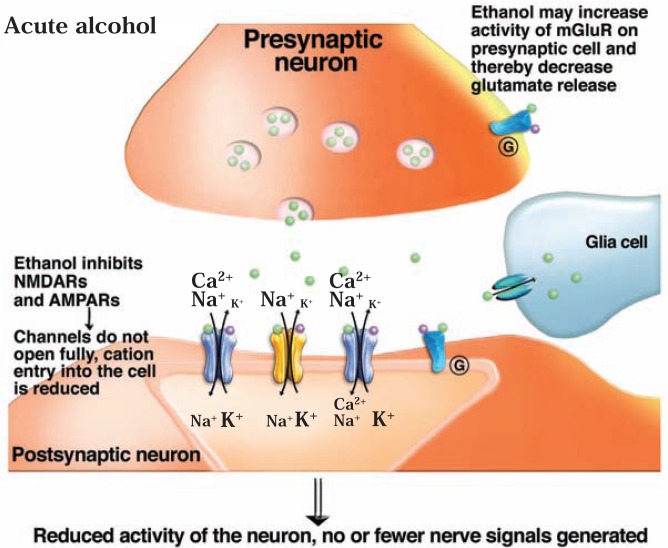
Actions of the brain’s glutamate system. In the presence of alcohol (ethanol, purple circles), the activity of the *N*-methyl-d-aspartate receptors (NMDARs) and α-amino-3-hydroxy-5-methylisoxazole-4-proprionic acid receptors (AMPARs), is inhibited, reducing cation entry into the cell. As a result, the activity of the neuron is reduced and no or fewer nerve signals are generated. For further information, see legend to figure 2A.

**Figure 2C:**
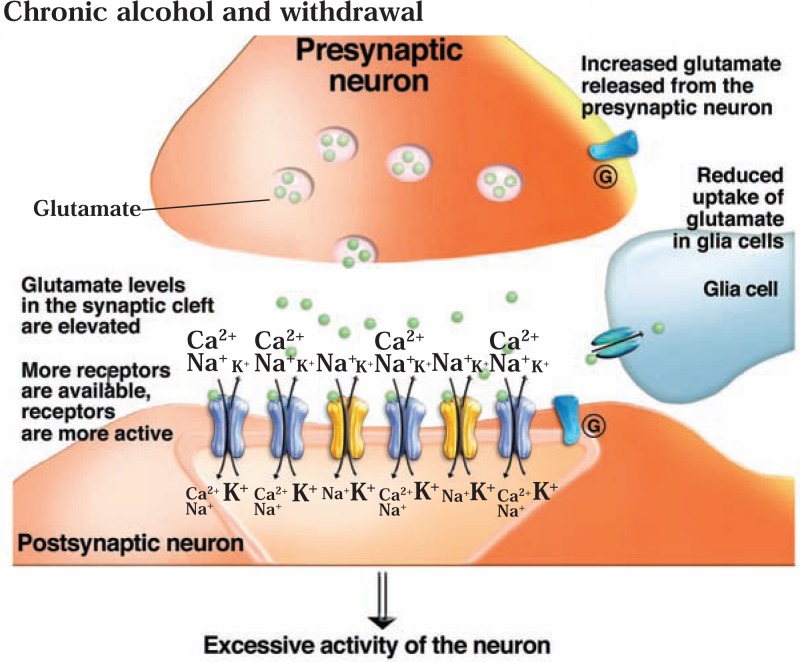
Actions of the brain’s glutamate system. After chronic alcohol exposure and during withdrawal, glutamate release at the synapse is enhanced and the number of synaptic *N*-methyl-d-aspartate receptors (NMDARs) and α-amino-3-hydroxy-5-methylisoxazole-4-proprionic acid receptors (AMPARs) is increased. As a result, glutamate induces excessive activity of the postsynaptic neuron. For further information, see legend to figure 2A.

**Figure 5A:**
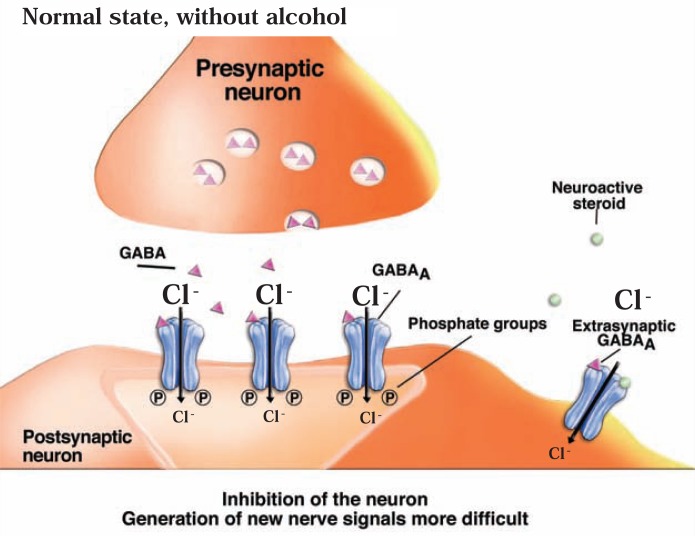
Actions of the brain’s γ-aminobutyric acid (GABA) system. GABA acts in part through GABA_A_ receptors, which serve as ion channels for chloride ions (Cl^−^). Greater influx of Cl^−^ into the neuron makes it more difficult for the cell to generate a new nerve impulse.

**Figure 5B:**
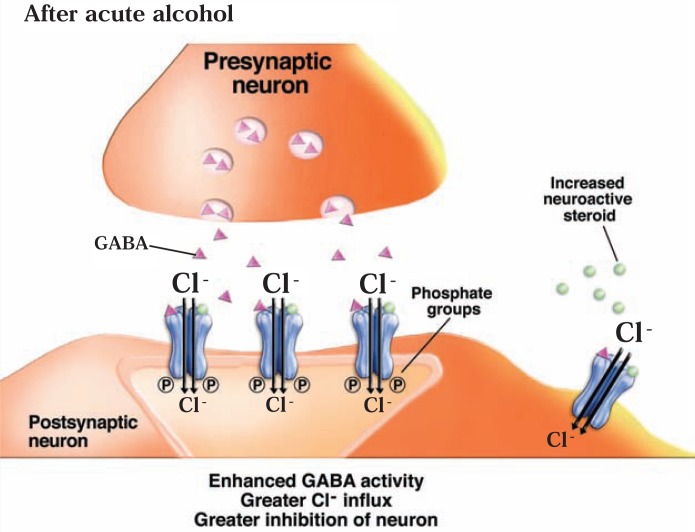
Actions of the brain’s γ-aminobutyric acid (GABA) system. In the presence of ethanol, GABA activity is enhanced, resulting in greater Cl^−^ influx into the postsynaptic neuron and, consequently, greater inhibition of the neuron. (For more information, see legend to figure 5A.)

**Figure 5C:**
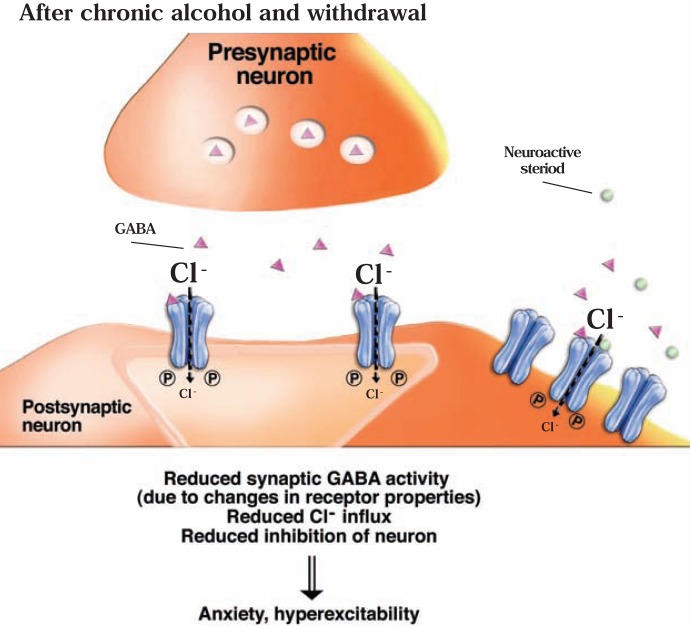
Actions of the brain’s γ-aminobutyric acid (GABA) system. After chronic alcohol exposure and during withdrawal, GABA activity at the synapse is reduced, leading to reduced inhibition of the postsynaptic neuron. This results in development of anxiety and hyperexcitability. (For more information, see legend to figure 5A.)

## Footnotes

1 These behaviors (which can occur in the presence or absence of physiological dependence) include drinking more alcohol than intended, unsuccessful efforts to reduce alcohol drinking, giving up other activities in favor of drinking alcohol, spending a great deal of time obtaining and drinking alcohol, continuing to drink alcohol in spite of adverse physical and social effects, and the development of alcohol tolerance.

2 For a definition of this and other technical terms, see the Glossary, pp. 345–347.

3 Although there is no specific alcohol receptor, certain “receptive elements” (e.g., receptor proteins) have been described that are affected by low concentrations of ethanol (Tabakoff and Hoffman 1983).

4 The affected brain regions include the prefrontal cortex and a group of structures known as the extended amygdala, including the central nucleus of the amygdala and the bed nucleus of the stria terminalis (BNST).

5 These receptors are named after synaptic substances that can interact with and activate them.

6 NR3A and NR3B receptor subunits are widely distributed in brain. The presence of NR3 subunits in a receptor reduces activity of NR1/NR2-containing receptors, and the combination of NR1 and NR3 subunits forms an excitatory glycine receptor. The role of NR3 in actions of ethanol on NMDA receptors has not been well studied.

7 An agonist is a substance that activates a receptor. The affinity is a measure of how easily and tightly a substance binds to a receptor.

8 High concentrations of ethanol inhibited the normal responses to mGluR5 activation in cells into which the receptors had been artificially introduced (i.e., in a heterologous cell system) (Minami et al. 1998).

9 To measure discriminative stimulus properties of alcohol, an animal is trained to give a certain response when it receives alcohol. If it gives the same response when it receives another compound, such as an iGluR antagonist, this indicates that the antagonist produces an effect that “feels” like alcohol to the animal.

10 An antagonist is a substance that inhibits or blocks the actions of another substance.

11 A ligand is any substance that specifically binds to a receptor or other molecule.
